# Intellectual disability genomics: current state, pitfalls and future challenges

**DOI:** 10.1186/s12864-021-08227-4

**Published:** 2021-12-20

**Authors:** Nuno Maia, Maria João Nabais Sá, Manuel Melo-Pires, Arjan P. M. de Brouwer, Paula Jorge

**Affiliations:** 1 Centro de Genética Médica Jacinto de Magalhães (CGM), Centro Hospitalar Universitário do Porto (CHUPorto), Porto, Portugal; 2 Unit for Multidisciplinary Research in Biomedicine (UMIB), Institute of Biomedical Sciences Abel Salazar (ICBAS), and ITR - Laboratory for Integrative and Translational Research in Population Health, University of Porto, Porto, Portugal; 3 Serviço de Neuropatologia, Centro Hospitalar e Universitário do Porto (CHUPorto), Porto, Portugal; 4 Department of Human Genetics, Donders Institute for Brain, Cognition and Behaviour, Radboud University Nijmegen, Nijmegen, The Netherlands

**Keywords:** Neurodevelopmental disorders, Intellectual disability, Massive parallel sequencing, Variant filtering, Variant prioritization, Animal and cellular modelling, Genome editing

## Abstract

Intellectual disability (ID) can be caused by non-genetic and genetic factors, the latter being responsible for more than 1700 ID-related disorders. The broad ID phenotypic and genetic heterogeneity, as well as the difficulty in the establishment of the inheritance pattern, often result in a delay in the diagnosis. It has become apparent that massive parallel sequencing can overcome these difficulties. In this review we address: (i) ID genetic aetiology, (ii) clinical/medical settings testing, (iii) massive parallel sequencing, (iv) variant filtering and prioritization, (v) variant classification guidelines and functional studies, and (vi) ID diagnostic yield. Furthermore, the need for a constant update of the methodologies and functional tests, is essential. Thus, international collaborations, to gather expertise, data and resources through multidisciplinary contributions, are fundamental to keep track of the fast progress in ID gene discovery.

## Background

Neurodevelopmental disorders (NDDs) are clinically defined as “a group of conditions with onset in the developmental period (…) characterized by developmental deficits that produce impairments of personal, social, academic, or occupational functioning” [*Diagnostic and Statistical Manual of Mental Disorders, 5th Edition* – DSM-5]. Intellectual disability (ID), formerly known as “mental retardation”, is an incomplete mental development, leading to a substantial limitation in general mental abilities, intellectual functioning, adaptive behaviour and function skills, in comparison with individuals of the same age, gender and social-cultural background [1]. These limitations can be observed in many domains such as communication, personal care, self-governance, functional academic skills, among others [1–3].

ID can appear as an isolated feature (non-syndromic ID, NSID), or associated with facial dysmorphic features, other morphological anomalies, multisystemic disorders (syndromic ID, SID) [4] or multiple neuropsychiatric and/or neurobehavioral problems, such as autism or epilepsy, and neuromuscular features, e.g. ataxia, spastic paraplegia, sensory or motor neuropathy, and muscular dystrophy [5–7]. Previously, ID classification was based on intelligence quotient (IQ) scores: mild (IQ 50–69, 85.0% of ID cases), moderate (IQ 35–49, 10.0% of ID cases), severe (IQ 20–34, 3.5% of ID cases) and profound (IQ < 20, 1.5% of ID cases) [1, 8–11]. Nowadays, ID diagnostic criteria include (i) deficits in intellectual functioning confirmed by clinical evaluation and standard IQ testing; (ii) deficits in adaptive functioning that results in failure to meet developmental and sociocultural standards for personal independence and social responsibility; and (iii) onset of deficits during the developmental period. The severity of ID is based on the level of adaptive functioning deficits of an individual in the conceptual, social and practical domains, which determines the level of support needed [1]. Under the age of 5 years, the term Global Developmental Delay (GDD) is used [2, 12, 13]. GDD is characterized by the failure to accomplish developmental milestones expected for a given age range, in two or more of the above-mentioned domains, including gross or fine motor skills, speech and language, cognition, personal-social and activities of daily living. ID and GDD are evaluated and clinically followed by the same medical specialties, in particular in paediatric clinics, psychiatry, neurology/epilepsy, and rehabilitation medicine clinics [14]. Of note, not all children with GDD will show ID in adulthood [15].

ID affects between 1 and 3% of individuals worldwide, although with some regional differences [16]. Mild ID is believed to affect 0.7–1.3% of the general population [17], while severe and profound ID have an estimated prevalence of less than 0.5%. ID represents an important public health problem, affecting families and the society, being a burden to the health systems with direct costs estimated in 43.3 billion euro per year in Europe [18]. Non-genetic or environmental factors, such as socio-cultural determinants and infections, can contribute to ID, although the majority of severe or profound ID are known to have a monogenetic origin [2, 7, 19, 20].

Technological advances in the last decade, led to the identification of novel ID genes, bringing new insights into the ID molecular diagnosis, and the underlying biological mechanisms [6]. Establishment of the ID genetic aetiology is mandatory for proper diagnosis, prognosis and disease management, assuming a key role in genetic counselling. Based on disease recurrence risk and the availability of a specific preimplantation or prenatal test, couples can be offered planning in future pregnancies [21]. Currently, ID is rarely treatable but molecular diagnosis is crucial to guide patients and families in the process of disease acceptance and expectations adjustment allowing the liaison with patient organisations and associations. The ragbag of ID classifications, diagnostic methodologies and functional studies demand constant update and systematization to improve ID diagnostic and investigational strategies. Here, we propose to review seminal works in ID particularly focusing on massive parallel sequencing applications and functional validation of genetic variants, aiming at guiding ID diagnostic investigation.

## Intellectual disability is genetically and clinically extremely heterogeneous

Genetic diagnosis of ID can be dated back to 1959 with the identification of trisomy 21 in Down syndrome [22], still being the most frequent chromosome disorder and the most common single cause of ID [23]. Conventional cytogenetics, namely karyotyping and fluorescence in situ hybridization (FISH), allow the identification of numeric and structural chromosome abnormalities, which are responsible for about 15% of ID [24]. Recurrent microdeletions and microduplications have been identified by chromosomal microarray analysis (CMA), in patients affected with ID-related disorders, including Williams, DiGeorge, Prader-Willi, Angelman, Wolf-Hirschhorn or Cri du Chat syndromes [6, 25]. DNA copy-number variants (CNVs) containing few to hundreds of genes, have increasingly been identified as ID causes [26]. CNVs, occur mostly de novo, and are responsible for about 10–14% of ID cases [26–29]. Research studies in cohorts of patients carrying recurrent CNVs allowed the identification of new disease and dosage sensitive dominant genes [30, 31].

Regarding monogenic ID cases, most are caused by single nucleotide variants (SNVs), and small insertions or deletions (indels), in genes that code for proteins that operate in key biological processes such as neurogenesis, synaptogenesis or synaptic plasticity. Development of a DNA sequencing method, the Sanger sequencing in 1975 [32], and further automatization and commercialization in the 1980’s, were key for the detection of this type of variants [33–35].

Non-Mendelian ID disorders are a challenge in diagnosis, genetic counselling and recurrence risk estimation. A special group are those caused by dynamic mutations occurring in tri, tetra and pentanucleotide repetitive regions. The first report of ID pathogenic variants caused by repeat expansions occurred in 1991. This study described the identification of a trinucleotide repetitive region, a CGG repeat tract located at the 5′ untranslated region of FMRP translational regulator 1 gene (*FMR1*) implicated in Fragile X syndrome (FXS) [36]. FXS is the most common cause of inherited ID, and despite being identified three decades ago, there is no effective treatment and knowledge on disease mechanisms is scarce [37]. To date, more than 40 inherited diseases affecting the central nervous system, have been identified [38–42].

Also, DNA methylation or DNA imprinting, well-known epigenetic disease mechanisms, do not follow a Mendelian inheritance pattern [43]. Imprinting diseases are implicated in ID, growth impairment, development and metabolism defects, associated with disturbance of the regulation, dosage and genomic sequence of imprinting *loci* [44]. The identification of consistent and significant methylation aberrations in multiple unrelated but phenotypically similar patients [43, 45, 46] is still challenging. The expression pattern of imprinted genes is monoallelic and parental origin dependent [47]. To date there are eight well-characterized imprinting disorders: Prader-Willi [48], Angelman [49], Silver-Russell [50], Beckwith-Wiedemann [51], Temple [52] and Kagami-Ogata syndromes [53], Transient Neonatal Diabetes [54] and Pseudohypoparathyroidism type 1B [55].

Another group of heterogeneous non-Mendelian genetic diseases are those caused by pathogenic variants in the mitochondrial genome (mtDNA), also known to be involved in ID [56]. Mitochondrial disorders are characterized by a deficient oxidative phosphorylation, with an estimated prevalence among adults of 2.9 cases per 100,000 individuals and 9.6 cases per 100,000 individuals, respectively caused by nuclear or mtDNA mutations [57]. Approximately 1 in 200 healthy individuals carry a pathogenic variant in mtDNA with low levels of heteroplasmy, with implications in the offspring [58]. Leigh syndrome caused by molecular defects in nuclear and mtDNA genes, and Mitochondrial DNA Depletion syndrome 4A (Alpers syndrome), are two examples of childhood-onset mitochondrial neurodegenerative disorders [59, 60].

The large genetic heterogeneity, intrinsic to ID-related disorders, as well as the absence of a specific inheritance pattern, especially when there is only one affected family member, can hamper the selection of the gene to target. To interrogate a large number of genes in a single step, tackling the majority of ID causes, including SNV, indels, CNVs and even structural chromosome abnormalities, the development of the genome-wide sequencing approaches, such as massive parallel sequencing, was essential.

## Massive parallel sequencing - a milestone towards ID-gene identification

Massive parallel sequencing commonly named next generation sequencing (NGS) is a fast, accurate, efficient and cost-beneficial screening strategy, representing a milestone in novel ID genes identification [61, 62]. Non-targeted NGS, a “genotyping driven” gene identification approach, unveiled the complexity of genotype-phenotype correlations, especially in heterogeneous disorders, where pathogenic variants in some ID-related genes can be implicated in “atypical” phenotypes [63, 64]. For instance, variants in *CHD2*, *SETD2* and *SLC6A1* genes are known to cause autism in some cases and severe ID without autistic features in others [27]. With the use of reverse phenotyping, clinicians return to patients to validate or infirm a molecular result even in cases of rare genetic occurrences. Describing new features associated with well-known phenotypes expanded the phenotyping spectrum of a given gene/disease, impacting ultra-rare disorders with atypical phenotypes [65].

**Fig. 1:**
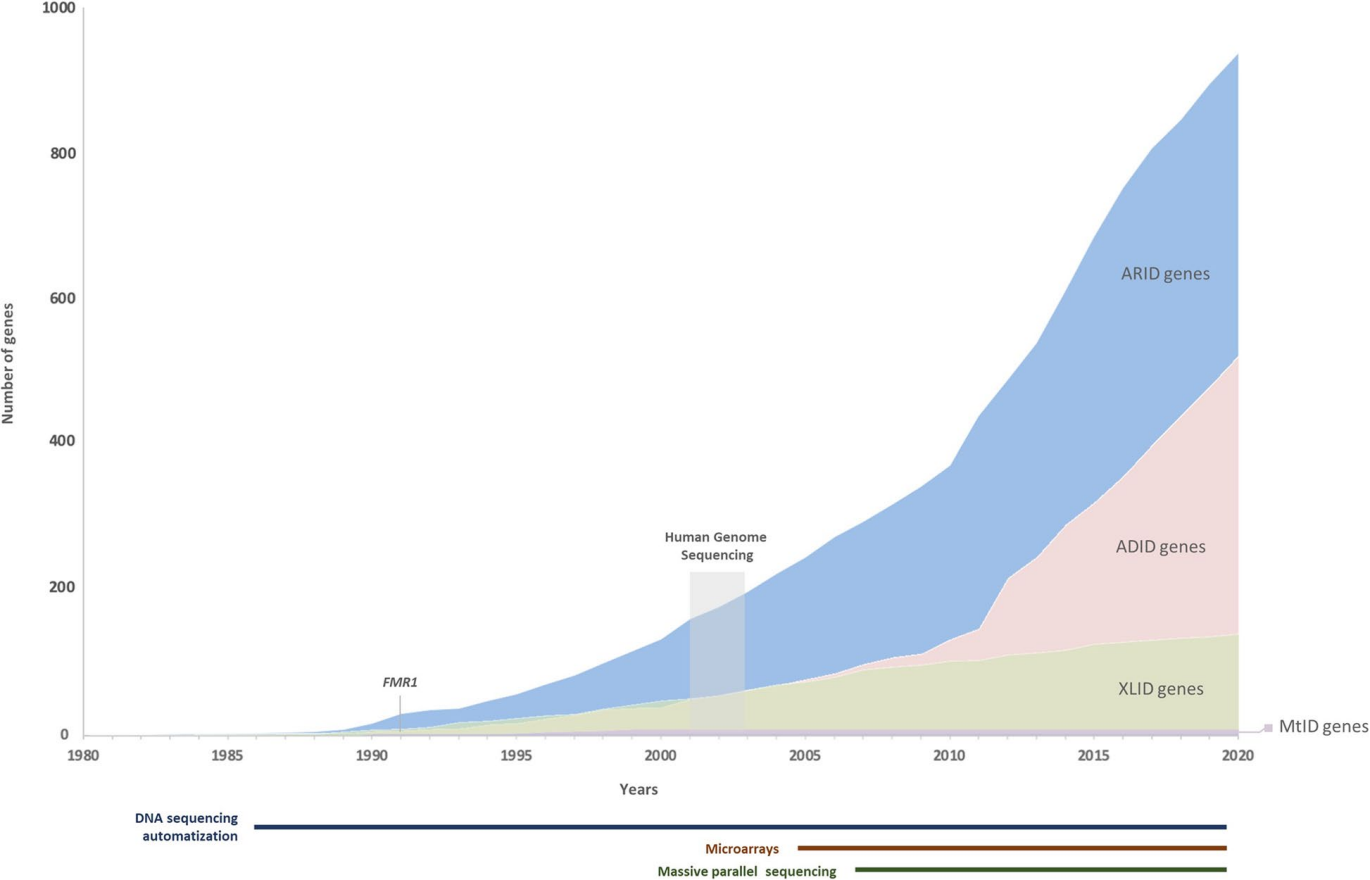
ID genes identified over the time. ID – intellectual disability; ARID – autosomal recessive intellectual disability; ADID – autosomal dominant intellectual disability; XLID – X-linked intellectual disability; MtID – mitochondrial intellectual disability. Reproduced from Vissers et al. [6] and updated with information from SysID database [4]

The first study using exome sequencing (ES) to uncover the genetic basis of Miller syndrome, a monogenic disorder, was published in 2010 [66]. In the last decade, new genes were rapidly associated with other autosomal dominant syndromes [6] and the number of autosomal recessive ID (ARID) genes more than doubled [67, 68]. Concurrently, more than 2500 ID genes, were identified, including primary and candidate genes (Fig. 1) [4].

**Fig. 2:**
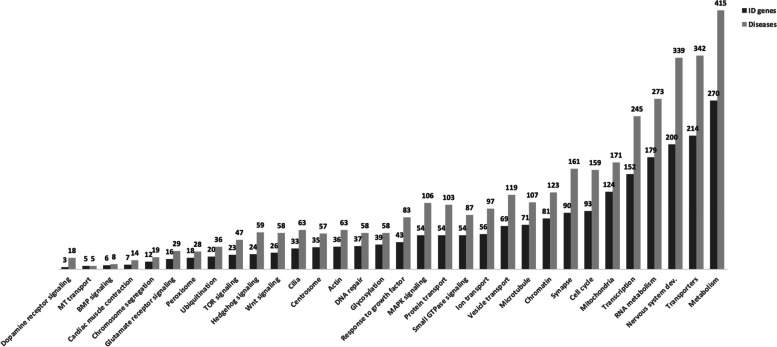
ID genes and diseases according to the corresponding ontology. Number of genes (dark grey) and related diseases (light grey) grouped by the biological pathway implicated. MT – mitochondrial; BMP – Bone morphogenetic protein; TOR – Target of rapamycin. Adapted from SysID database [4]

According to the SysID database, there are 1500 primary ID genes, causing 1797 ID related disorders, and 1248 ID candidate genes. ID related genes can be gathered based on their ontology, or biological function (Fig. 2). The gene ontology-based analysis shows the large heterogeneity of ID, as well as the biological pathways involved. Gene cluster analysis shows 270 genes and 415 diseases associated with metabolism [4]. Phenylketonuria and galactosemia, caused by molecular deficits in *PAH* and *GALT* genes respectively, are examples of such disorders, representing 1–5% of ID causes [69]. A significant number of ID genes/diseases are also involved in transport (214/342), nervous system development (200/339), RNA metabolism (179/273) and transcription (152/245) [4].

### Common features: from library preparation to sequencing reactions

**Fig. 3:**
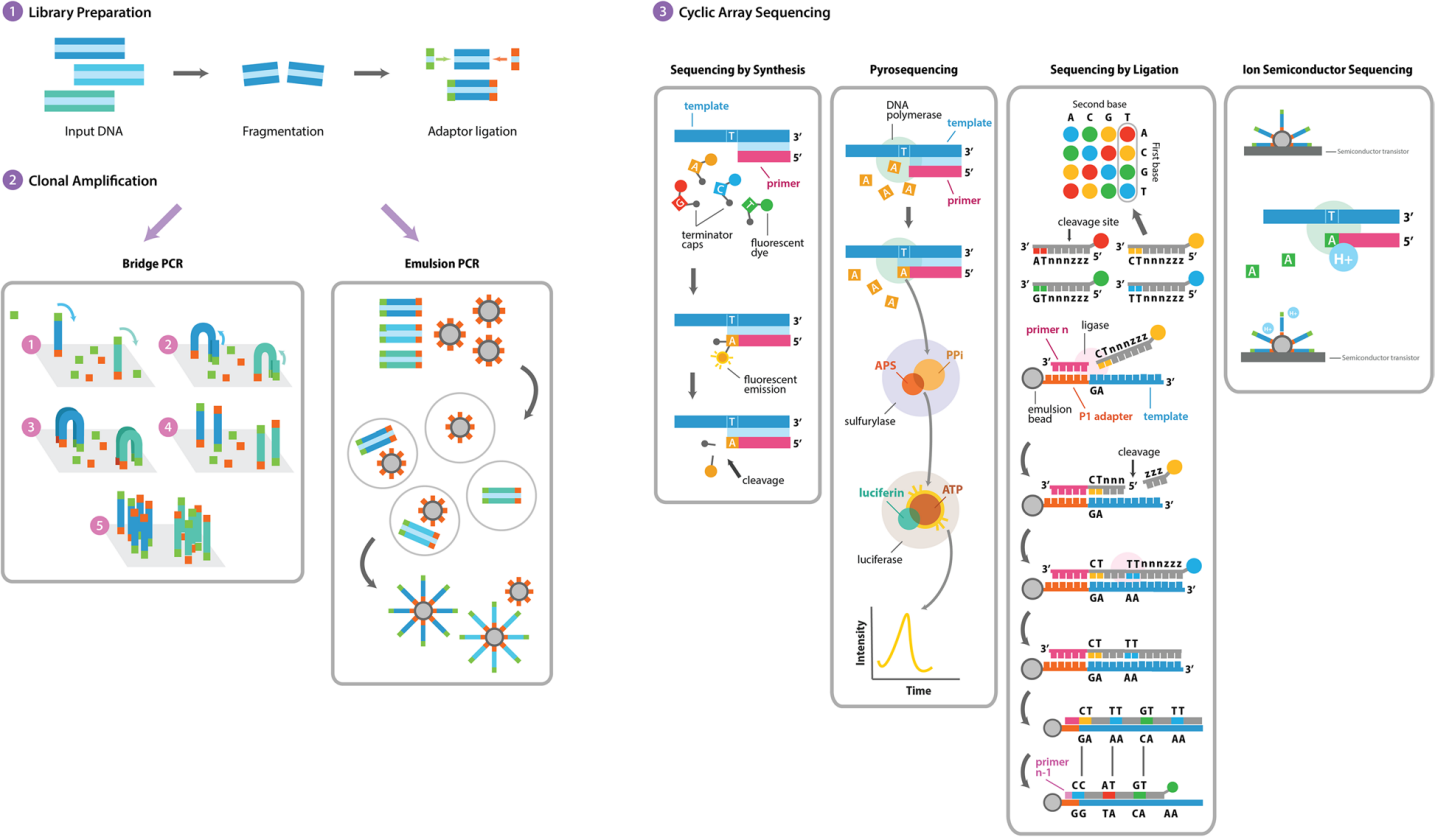
Overview of the NGS techniques. Schematic representation of the common features (1 and 2) and different particularities (3). APS – Adenosine 5′ phosphosulfate; PPi – Inorganic pyrophosphate; ATP – Adenosine triphosphate; P1 – Primer 1. Reproduced by permission of Applied Biological Materials Inc. (abm)

Four sequencing platforms sharing common basic features, such as library preparation and template generation, were hitherto developed. Sequencing reactions are intrinsic to each methodology and the signal resulting from the amplification is obtained by fluorescence, light or ionic potential modification, depending on the underlying principle: sequencing by synthesis, pyrosequencing, sequencing by ligation and ion semiconductor sequencing (Fig. 3) [70, 71].

Sequencing by synthesis is based in a step-by-step incorporation of nucleotides attached to a single florescent molecule. The error rate is low, although increasing with the read length [72]. In pyrosequencing, a pyrophosphate molecule is released and light will be generated after a cascade of chemical reactions, following the polymerase incorporation of a nucleotide. This results in larger read lengths, but with high costs and high error rate over homopolymers of 6 or more nucleotides [73]. In ligation, the reaction is based in fluorescent 8-mer oligonucleotide probes, resulting in very short read lengths [74]. In ion semiconductor sequencing, the nucleotide sequence is determined by pH changes. Overall, this is the most cost-effective and time-efficient, despite the high error rate in large homopolymers [75].

### Targeted-NGS is effective on clinically recognizable forms of ID

Targeted-NGS (TNGS) has been largely used in ID diagnostic settings, either using panels of genes involved in common pathways, or by studying an entire chromosome. Najmabadi et al. [76] identified putative disease-causing variants in 78 out of 136 consanguineous families (57%), resulting in the identification of 50 candidate ARID genes and variants in known syndromic-ARID genes in 26 families. Tzschach et al. [77], sequenced 107 XLID genes in 50 patients with a suggestive XLID family history and in 100 sporadic ID patients, identified pathogenic variants in 13 (26%), and in five (5%) patients, respectively. Hu et al. [5] identified seven novel XLID genes: *CLCN4*, *CNKSR2*, *FRMPD4*, *KLHL15*, *LAS1L*, *RLIM* and *USP27X* and a previously characterized ID pathogenic variant in 74 families (18%), after sequencing 745 genes in 405 families. The diagnostic yield is biased to the targeted regions and influenced by the clustering of genetic errors, typically occurring in regions with high homozygosity due to inbreeding, such as in Iran [78–80]. In well characterized patients with dysmorphic, neurological or systemic features, TNGS low sequencing costs, high coverage, completeness and incidental findings low-rate, results in a decrease in the diagnostic turnaround time. As knowledge evolves, e.g. new disease associated genes are identified, updates are needed, which can be laborious, time-consuming and increase the TNGS costs [81–83].

### Exome-sequencing improves the diagnostic yield in syndromic NDDs

Exome sequencing (ES) has been shown to be a powerful, robust, and scalable methodology in ID diagnosis. Trio-ES analysis (i.e. proband and parents) led to the identification of a significant number of de novo variants in patients with sporadic ID [84]. De Ligt et al. [2] performed a trio-ES study in 100 families and identified 70 de novo variants in 53 patients, with an overall diagnostic yield of 53%. Rauch et al. [27] identified 87 de novo variants of which 16 in known ADID genes, in 45 out of 51 patients after a negative CNV screening. Considering the six loss-of-function variants, identified in six novel ADID genes and assumed to be pathogenic, a diagnostic yield of 88% is achieved. The Deciphering Developmental Disorders (DDD) study recruited families from all regional genetics services around the United Kingdom (UK) and Ireland. Around 2000 families with undiagnosed developmental disorders were included in the first year of the study, increasing to 8000 within 3 years. After genome-wide microarray and trio-ES studies, focusing 1133 complete trio-families, de novo and segregating variants in known developmental disorder genes were identified, representing a diagnostic yield of 27% [85]. In 2018, data were reanalysed in light of new molecular and clinical knowledge and a diagnosis was attained in further 454 families, representing a diagnostic yield of 40% [86]. In 2019, a meta-analysis gathering information on 30 NDDs studies published between January 2014 and June 2018 concluded that the ES yield for overall NDDs is 36%, isolated NDDs 31%, and syndromic NDDs 53 [87].

### Genome-sequencing: a complete approach

Genome sequencing (GS) provides homogeneous coverage, improving the detection of SNVs, CNVs, and balanced translocations [88], as well as the detection of mosaicism, when coverage depth is sufficient (e.g. a mean coverage of 130 ×) [80, 89]. In the Schluth-Bolard et al. [90] study, balanced chromosomal rearrangements with inversions and translocations were identified in three patients. Gilissen et al. [67] identified 84 de novo CNVs and 82 SNVs in a cohort of 50 patients, previously undiagnosed after ES, reaching a conclusive diagnosis in 21 patients (42%). These authors estimate that the cumulative diagnostic yield of GS was 62%, including de novo SNVs (39%), de novo CNVs (21%) and recessive variants (2%), based on previously published data with large cohorts [67]. In a cohort of 244 ID/developmental delay (DD) children, Bowling et al. [91] identified 44 pathogenic and 10 likely pathogenic SNV/indel variants, 5 pathogenic and 1 likely pathogenic CNVs, resulting in a diagnostic yield of 25%. Wang et al. [92] tested whole genome low-coverage sequencing to detect CNVs, and medical exome sequencing (MES), i.e. exome analysis of known ID disease-causing genes, to identify SNVs, in 95 patients with a negative CNVs screening. Nineteen pathogenic CNVs in 16 patients (17%), and ten pathogenic SNVs in 8 patients (8%) were found [92]. GS is the most comprehensive genetic test, as it interrogates all the genome [67], however, improvements in the bioinformatics algorithms for variant detection and interpretation are needed. Together with the decrease of the associated costs, are crucial for the routine implementation of GS in diagnostic settings [93].

### Variant filtering

**Fig. 4:**
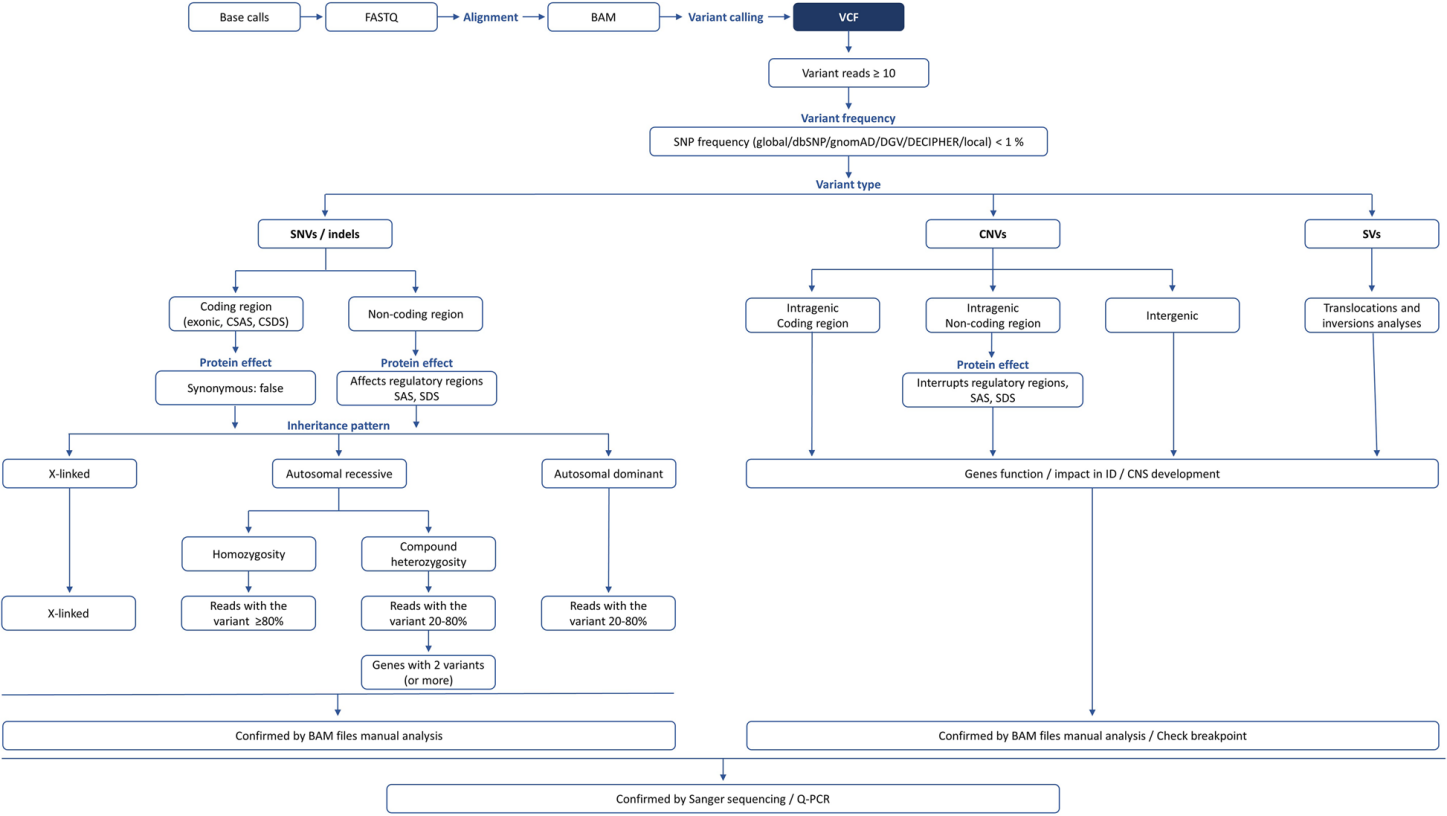
Variant filtering flowchart. SNP – single nucleotide polymorphism; DGV – database of genomic variants; SNVs – single nucleotide variants; CNVs – copy number variants; SVs – structural variants; CSAS – canonical splicing acceptor site; CSDS – canonical splicing donor site; SAS – splicing acceptor site; SDS – Splicing donor site; Q-PCR – quantitative PCR

Massive parallel sequencing raw data is standardly generated in the FASTQ format. The files contain the identification, sequence and sequence identifier, and quality values of each sample [94]. Reads are usually mapped into the hg19/GRCh37 or GRCh38 versions of the human reference genome, and the alignment results are typically reported in binary alignment map (BAM) format. BAM files contain information on the possible location of each read in the human genome [95]. After sequence alignment, variant calling will identify differences between the reads sequence and the reference genome. Variants are usually reported as variant call format (VCF) file. VCF files are composed of several lines, each corresponding to a genomic position [96]. Sophisticated algorithms as used to screen the information generated after genome sequencing with inherent data storage and interpretation issues. Due to the intrinsic ID heterogeneity, the use of guidelines are important. Figure 4 represents a simplified workflow to guide variant filtering.

#### Variant coverage

Variants occurring in 20% of the reads, with a minimum coverage of ten, should be considered to reduce the prioritization of sequencing artefacts [93, 97]. Nevertheless, variants occurring in less than 1% of the reads can be identified, when sufficient coverage is attained (e.g. 30–60 x for genome) [97]. Rohlin et al. [98] study suggest a high mosaicism detection rate when compared with other molecular techniques, but dependent on coverage levels. Jamuar et al. [99] identified mosaic pathogenic variants, the majority of which were undetected by conventional Sanger sequencing, in peripheral blood DNA from patients with brain malformations, using high-coverage sequencing target panels.

#### Variant frequency

Variants causing uncommon and severe conditions usually are rare among the general population, and therefore variants with a frequency ≥ 1% (based on SNPs – Ensembl [100], dbSNP [101] and gnomAD [102], for SNVs and small indels, Database of Genomic Variants (DGV) [103] or DECIPHER [104] in case of CNVs, and other in-house databases) can be excluded from further analysis. Exceptions are those involved in rare oligogenic diseases that can exceed 18% [105] and common variants (minor allele frequency, MAF ≥ 5%) generally located in non-coding regions [106]. Niemi et al. [107] studied a cohort of 6987 children with severe NDDs and showed that inherited common variants were responsible for 7.7% of risk variance. Databases have emerged focusing on non-coding regions regulatory elements, such as CODE (http://www.encodeproject.org) [108] and Orion (http://www.genomic-orion.org) [109].

#### Variant percentage among reads

The inclusion of the putative ID Mendelian inheritance in the filtering strategy and variant prioritization may help to organize information and to reduce the number of candidate variants [110, 111]. For instance, homozygous variants are often associated with consanguinity, and therefore more common in inbred populations, and ID sporadic cases are frequently caused by autosomal dominant de novo pathogenic variants [78]. Ancestry is therefore relevant information to consider before prioritization [78, 79]. Homozygous variants usually show a > 80% variant allele frequency (VAF), whereas compound heterozygous variants show a VAF varying from 20 to 80% among reads.

#### Variant review

Candidate variants should be reviewed by manual analysis, using a suitable software such as the Integrative Genomics Viewer (IGV) [112]. Although still debatable, gold standard methodologies might be used to confirm variants [113, 114], such as Sanger sequencing for SNVs, and genomic quantitative PCR (Q-PCR) for CNVs.

## Variant deleteriousness categorization

**Fig. 5:**
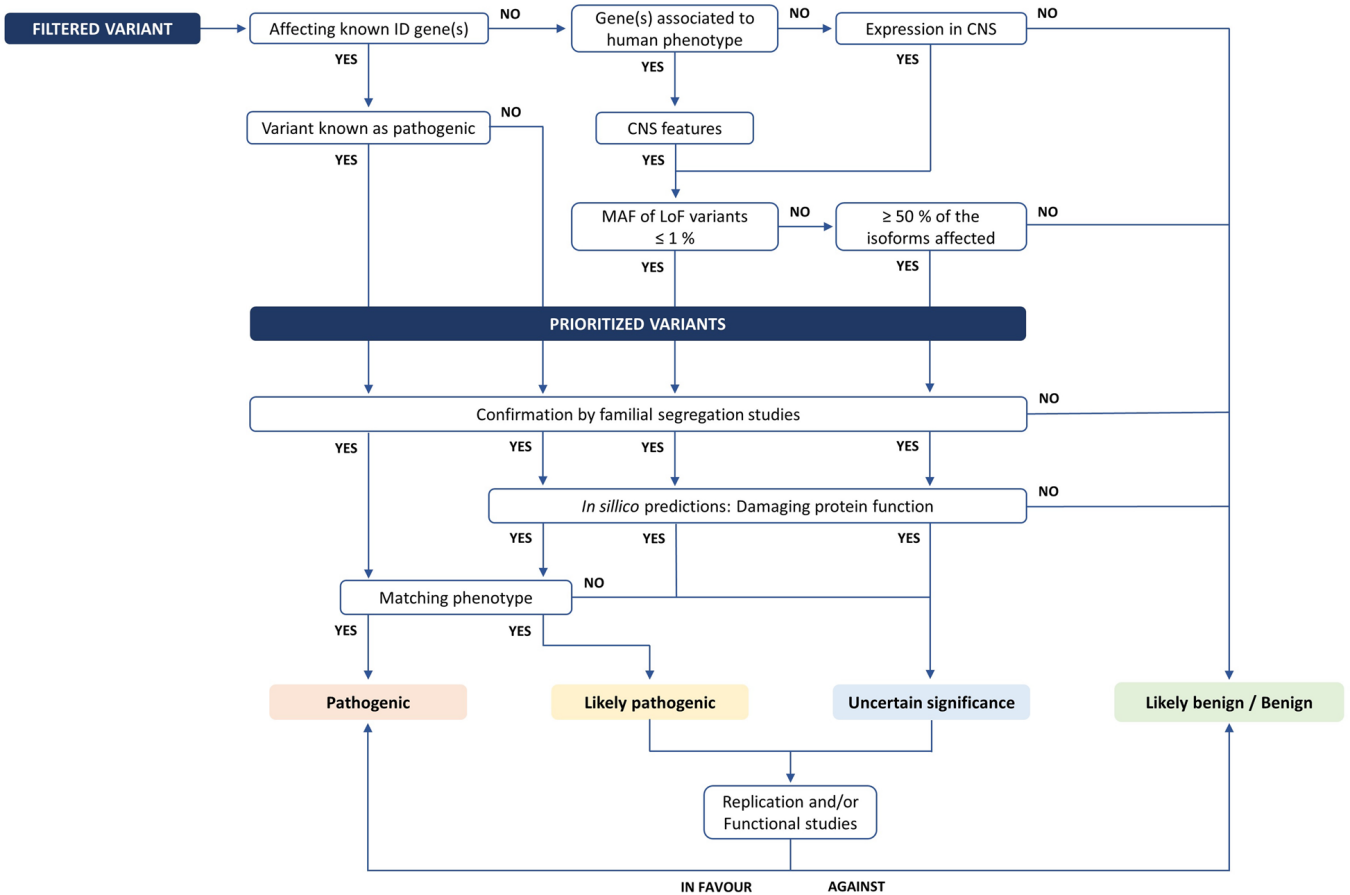
Variant classification flowchart. ID – intellectual disability; CNS – central nervous system; MAF – minor allele frequency; LoF – loss of function. Adapted from Schuurs-Hoeijmakers et al. [116]

We suggest sequential steps for accessing the functional impact of variants in ID, towards variant classification in five categories: pathogenic, likely pathogenic, uncertain significance, likely benign, and benign, according to the American College of Medical Genetics and Genomics (ACMG) and the Association for Molecular Pathology (AMP) recommendations (Fig. 5) [115].

Known pathogenic variants in well-recognized ID genes, based on the data published at ClinVar [117], ClinGen [118], OMIM [119, 120], and SysID [4] databases, should be first prioritized. Other aspects should then be considered: (i) implication in other disorders, with central nervous system (CNS) impairment; (ii) levels of expression in CNS/brain, (iii) interaction with other proteins implicated in ID, or (iv) biochemical function similarity with other ID genes (Table 1). Variants predicted to seriously disrupt the protein function (e.g. Loss of function, LoF) with a MAF of ≤1%, and its presence in > 50% of isoforms, should follow. When available, familial studies are used to confirm the segregation of each suitable candidate variant with the phenotype.

**Table 1:** Bioinformatic analysis databases and tools

	Databases and bioinformatics tools	Description	URL	References
Clinical significance of genomic variants	ClinVar	Relationships between human genomic variations and phenotypes, with supporting evidence	https://www.ncbi.nlm.nih.gov/clinvar	[117]
	ClinGen - Clinical Genome Resource	Clinical relevance of human genes and variants	https://clinicalgenome.org	[118]
Human genes and phenotypes	Online Mendelian Inheritance in Man (OMIM)^a^	Human genes and genetic phenotypes, Mendelian disorders and phenotype-genotype correlations	https://www.omim.org	[119, 120]
	Systems Biology Approaches to ID (SysID)	ID genes description, disease related information and pattern of inheritance, clinical information, protein-protein interactions, specific biological functions and *Drosophila* orthologues and identified phenotypes	https://www.sysid.dbmr.unibe.ch	[4]
	The Human Phenotype Ontology (HPO)	Standardized vocabulary of phenotypic abnormalities encountered in human disease	https://hpo.jax.org/app	[121]
Gene expression patterns	EMBL-EBI Expression Atlas	Gene and protein expression across species and biological conditions, *e. g.* different tissues, cell types, developmental stages and diseases among others	https://www.ebi.ac.uk/gxa/home	[122]
	The Common Fund’s Genotype-Tissue Expression Program (GTEx Program)	Tissue-specific gene expression and regulation	https://www.gtexportal.org/home	[123]
Protein interactions	The Universal Protein Resource (UniProt)	Protein sequence and biological functional information	https://www.uniprot.org	[124]
	IntAct Molecular Interaction Database	Molecular interaction data	https://www.ebi.ac.uk/intact/	[125]
Gene/protein functions	GeneCards	Genomic, proteomic, transcriptomic, genetic and functional information on all known and predicted human genes	https://www.genecards.org	[126]

^a^Also provides information regarding gene/protein functions

### In silico causality prediction

Particularly in missense variants, causality ascertainment is challenging [27], with an accuracy of about 80%, despite the improvement in the in silico pathogenicity predictions tools [78]. In Rauch et al. [27] work, two *NAA10* variants were classified as pathogenic based on the expected protein effect and patient’s phenotype, yet predicted as benign using in silico tools. Putative splicing effect can be screened using tools such as SpliceSiteFinder-like (normal score threshold ≥70 for SDS and SAS) [127], MaxEntScan (normal score threshold ≥0 for SDS and SAS) [128], NNSPLICE (normal score threshold ≥0.4 for SDS and SAS) [129] or GeneSplicer (normal score threshold ≥0 for SDS and SAS) [130] and Combined Annotation Dependent Depletion cut-off ≥15 (CADD, http://cadd.gs.washington.edu/score) [131] to predict gene disruption.

### Replication studies

Gather unrelated patients with a similar phenotype and carrying putative deleterious variants in the same gene, i.e. replication, is crucial to identify new ID genes. Nevertheless, assembly patients that comply to these characteristics is problematic, particularly in rare ID syndromes. To overcome this bottleneck several open-access online platforms allow data sharing:

(i) GeneMatcher (https://genematcher.org) [132, 133],
(ii) Human Disease Genes website series (http://humandiseasegenes.info) [134],
(iii) PhenomeCentral (https://www.phenomecentral.org) [135],
(iv) Leiden Open Variation Database (LOVD, https://www.lovd.nl) [136],
(v) Clinvar (https://www.ncbi.nlm.nih.gov/clinvar) [117], and
(vi) Solve-RD - solving the unsolved rare diseases (https://solve-rd.eu) [137], among others.

### Model organisms

In vivo and in vitro studies are particularly important to disclose the deleteriousness of ambiguous or novel variants as well as to implicate new genes in ID phenotypes. The implementation of ID functional studies, using model organisms or patient-derived tissues or cells, is however, complex in a diagnostic facility [78]. Since the 1980s and 1990s, models have been used to understand the mechanisms of monogenic ID disorders, as orthologous genes are involved in evolutionary conserved biological processes [138]. Simple organisms, with short life cycles, allowing genetic manipulation, can easily give insights into several biological processes [139]. Next, several model organisms and corresponding ID seminal studies will be described.

#### Yeast

Yeast has been considered a valuable ID model following the advances in “autophagy” knowledge, a mechanism compromised in neurological disorders [140]. *Saccharomyces cerevisiae*, with 23% of homology with human genes [141], shares particular evolutionary conserved key elements with neurons, e.g. budding or mating in yeast to neurite outgrowth or spinogenesis in neurons [142]. Yeast models were used to define (i) the function of septin in the differentiation and compartmentalization of neurons [143], (ii) the role of the MED12-complex in transcriptional regulation [144], and (iii) the mechanisms underlying mitochondrial disorders [145]. Furthermore, has been used to study aging mechanisms and age-associated neurodegenerative disorders (reviewed by Ruetenik et al. [146]).

#### *Caenorhabditis elegans*

The nematode *Caenorhabditis elegans* has also been largely used as a model for neurodevelopmental disorders [147]. With approximately 41% homology with human genes, a short life cycle, easy cultivation and accessibility to the entire nervous system structure [148]. *C. elegans* revealed to be a very valuable model to study crucial processes, such as cell birth and diversification, cell migration, morphogenesis and pathfinding, synapse formation, and neurite/synapse sorting maintenance and plasticity (reviewed by Rapti [147]). The use of *C. elegans* brought important insights into the human system nervous illness, such as epilepsy, autism spectrum disorder (ASD) and ID (reviewed by Bessa et al. [149]).

#### *Drosophila melanogaster*

Identification of conserved genes and pathways in *Drosophila melanogaster* (with 75% homology to human genes), goes back to the end of the 1970s [150, 151]. The genes involved in wings development and pattering contributed to the characterization of pathways and mechanisms responsible for skeletal and craniofacial abnormalities in humans [138]. *Drosophila* is a reference model in ID and ASD as the neuromuscular junction show structural, morphologic, and functional similarities to human synapses [152]. Allowing the study of subcellular events, such as synapses and dendritic complexity, neurotransmission and circuit connectivity, neuronal activity and physiology, brain morphology, and behaviour alterations such as learning and social interaction issues [153], makes *Drosophila* a valuable and complete model to understand those disorders. Some human genes do not have a homologue in *Drosophila*, where vertebrate models, such as zebrafish and mice, are useful.

#### Zebrafish

Zebrafish, with 70% of genomic content homology with humans [154], and similar CNS structures, such as the hippocampus, diencephalon, tectum and tegmentum, and cerebellum, has emerged as an important disease model but also to test potential therapeutic solutions [155]. Zebrafish has a short reproductive cycle, transparent embryos and larvae, easy access to the central nervous system [156], being used to recapitulate: (i) behaviour, such as hypoactivity and hyperactivity, hyperexcitability, impulsiveness, aggressiveness, circadian disturbances, and schizophrenia; (ii) cognitive, learning and memory deficits, and structural abnormalities; or (iii) physical, such as microcephaly, macrocephaly and microphthalmia, some of the neurodevelopmental disorders clinical features. Zebrafish has been widely used as model for ASD, attention deficit hyperactivity disorder (ADHD), ID and schizophrenia-like phenotypes (reviewed by De Abreu et al. [157]).

#### Mice

ID research, NDD investigation, including development of innovative therapies is anchored in mice studies, due to the similarity (90%) between both genomes [158]. Pivotal studies include: (i) biochemical alterations, such as Mecp2-related deficit in Gamma aminobutyric acid (GABA) and glutamate synthesis pathway [159], and the imbalance of brain metabolites in the hippocampus of *Fmr1* KO mice during the developmental period of synaptogenesis and early myelination [160], (ii) changes in synaptic morphology and function, such as *Syngap1* associated to early maturation of the spines [161], and decrease of dopamine auto receptors in *Mecp2* KO mice [162], and (iii) behavioural issues, such as social impairment, communication problems, repetitive behaviour and resistance to change in routine, cognition, memory, and learning.

### Patient-derived cellular models

The brain is an unavailable organ in live humans whereas post-mortem tissue gives information mostly on the end-stage of a disease, providing little contribution on early brain development or impairment [163]. ID genes are differently expressed during brain development and thus the impact of variants in such genes should be accessed at the suitable stage of maturity [164]. Cellular models to study monogenic ID disorders have emerged as an alternative to animal models [165], such as human-induced pluripotent stem cells (hiPSCs).

#### Human-induced pluripotent stem cells

hiPSCs differentiation allow generation of somatic cells, including human neurons at early developmental stages. Patient-derived fibroblast can be reprogramed into iPSCs using the “OSKM” factors (Oct3/4, Sox2, Klf4, and c-Myc), and then differentiated into highly pure populations of glutamatergic, GABAergic, dopaminergic, serotonergic or motor neurons, astrocytes, or oligodendrocytes, depending on the transcription factor used [163, 166]. The simultaneous culture of two or more cell types is possible allowing a physiological contextualization and recapitulation of the human biological systems [167]. hiPSC models have been used in ID-related disorders, such as Rett, Fragile-X, Dravet, Phelan-McDermid, Miller Dieker, Angelman, Prader-Willi, Timothy, Williams-Beuren and Lowe syndromes, Friedreich’s ataxia, Alexander and Pelizaeus-Merzbaucher diseases, primary microcephaly and X-linked adrenoleukodystrophy (reviewed by Sabitha et al. [168]). The duration of the procedure and the expertise needed, are some of the limitations [169]. Additionally, phenomena such as genetic instability and epigenetic alterations leading to changes in gene expression can occur during the reprogramming procedure, and hamper results interpretation [170]. Furthermore, hiPSCs do not recapitulate behavioural phenotypes, nor the influence of environmental factors or late-onset diseases due to their incomplete maturation [171].

#### Induced neurons

Induced neurons (iNeurons) have shown to be a promising alternative to hiPSCs, as they preserve the original somatic age-related epigenetic landscape. iNeurons resulting from differentiation of mouse embryonic fibroblasts using the transfection factors Ascl1, Brn2, and Myt 1 l (BAM pool) [172] were first developed in 2010. To overcome the need of an invasive sample collection, such as skin biopsy, Tanabe et al. [173] described a method to generate neurons by reprogramming blood nuclear cells (blood iNs). Nevertheless, the necessary co-culture with mouse glia convolutes the interpretation of the results, as these cells can distort neuronal morphologies [171].

### Genome editing using CRISPR platforms

Genome editing systems such as the Clustered Regularly Interspaced Short Palindromic Repeats (CRISPR) are indispensable tools in biological research [174, 175]. The key success in the CRISPR mechanism is the association of a RNA guide (gRNA) and Cas9 protein. While the gRNA, a 20-nt targeting sequence, recognizes DNA sites by base pairing, the Cas9 cleaves DNA, through double-strand breaks (DBS) induction, activating DNA repair mechanisms such as nonhomologous end joining (NHEJ) or homology-directed repair (HDR) [174, 176]. Several CRISPR/Cas9-based studies have been carried out in hiPSCs, showing their efficiency and potential (reviewed by Ben Jehuda et al. [176]). The use of CRISPR and hiPSCs simultaneously allows analysis in a donor-independent manner, overcoming the heterogeneity often observed in hiPSCs, due to the specific genetic background, epigenetic factors or other inter-individual differences [164, 168]. One of the limitations of CRISPR/Cas-9 editing system is the off-target effects i.e. Cas-9 binds and cleaves unintentional genomic sites [164, 177]. The “prime editing” combining Cas9 and a reverse transcriptase, allows genome editing without the double-strand DNA breaks collateral effect [178]. CRISPR interference (CRISPRi) and CRISPR activation (CRISPRa) have been developed as alternatives for previous genome editing platforms. The CRISPRi/a result from the fusion of the CRISPR technology with a dead nuclease (dCas9), allowing the repression/activation of gene expression at the transcriptional level [179]. These tools, so far eligible for Mendelian disorders, mandate recommendations and guidelines to ensure that human genome editing is used ethically and safely.

## Concluding remarks

Diagnostic approach in a medical genetic setting begins by the observation and categorization of the clinical features [180]. The Human Phenotype Ontology Project (HPO; https://hpo.jax.org/app/) terminology gathers a set of terms and codifications of signs, symptoms, and other phenotypic manifestations, contributing to an accurate phenotyping. By adopting this terminology, clinical data can be shared and integrated across the scientific and medical communities [121, 181], guiding geneticists towards the definition of the ID diagnosis strategy and molecular defect identification. While at this point genotype-phenotype correlation is complex, new ID classification systems have emerged. Kochinke et al. [4] developed a phenotype-based bipartite clinical classification system that interprets the phenotypic heterogeneity characteristic of monogenic ID. Recently, Biesecker et al. [182] suggested a syndrome definition based on the affected gene and phenotypic description. Using the ID gene *GLI3* as an example, a clear and simplistic description of several heterogeneous diseases would be *GLI3*-related Pallister-Hall syndrome or *GLI3*-related Greig cephalopolysyndactyly syndrome.

**Fig. 6:**
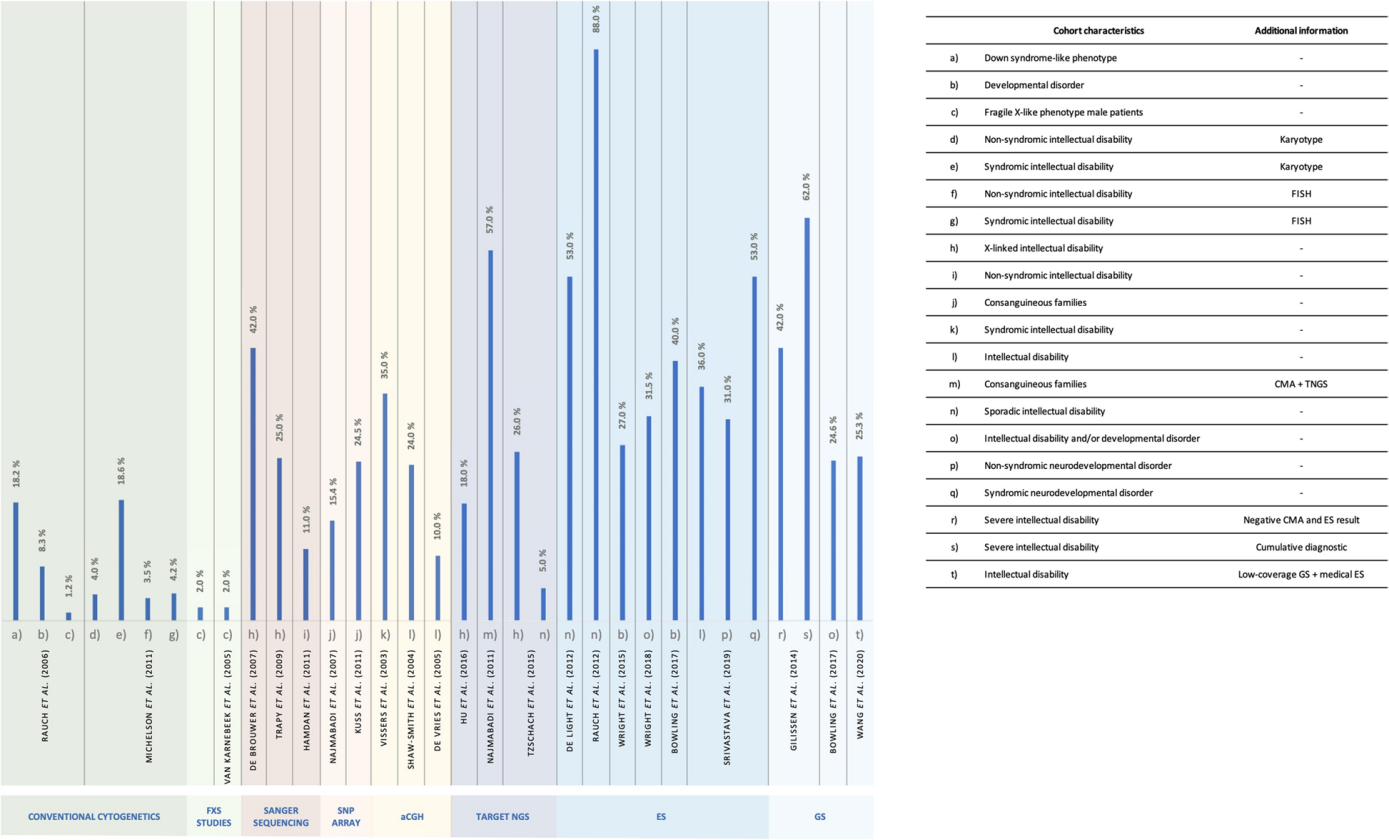
ID diagnostic yield. Rate of ID diagnosis in different studies, indicated by the name of the first author and the year of publication. Coloured rectangles correspond to the methodology used in each study [2, 5, 21, 24, 26–28, 67, 76, 77, 85–87, 91, 92, 186–192]. FISH - fluorescent in situ hybridization; CMA - chromosomal microarray; TNGS - Targeted-NGS; ES - exome sequencing; GS - genome sequencing

The literature indicates that high ID diagnostic yields are attained by applying the following sequential testing strategy using validated methods, after a detailed clinical evaluation: numeric and structural chromosomal abnormalities analysis, FXS testing, *MECP2* (females) and *PTEN* genes investigation (in the presence of ASDs with macrocephaly) [183], CNVs screening by CMA [184] and exome sequencing [87, 185]. As illustrated in Fig. 6, ID diagnostic yield depends on the technology used, the presence and variability of other clinical features and inheritance pattern (Fig. 6). De Brouwer et al. [186] demonstrated the importance of a deep, accurate and homogeneous phenotyping, after diagnosing 42% of patients with XLID. Najmabadi et al. [76], combined microarray analysis and massive parallel sequencing with a diagnostic yield of 57%, in consanguineous families. Nevertheless, caution is warranted when comparing data from different studies, and special attention should be drawn to the heterogeneity of the clinical descriptions and putative bias in patient ascertainment.

ID diagnosis strategy should also include systematic reanalysis of previously generated data, in light of the new knowledge [193, 194], e.g. databases update, novel disease genes identification, new clinical features and molecular information [195]. This is a clear advantage of the ES / GS over the TNGS. Reanalysis of ES data from 1133 children with severe developmental disorders and their parents, increased the diagnostic yield from 27% (2015) to 40% (2018) [85, 86].

To date, the ID diagnostic yield remains low, and the identification of previously undetected variants in non-coding regions by GS will clarify hitherto some molecularly undiagnosed ID cases. Moreover, the recent development of long-read sequencing (LRS), namely Single-molecule real-time (SMRT) sequencing, using PacBio sequencing (Pacific BioSciences, Menlo Park, CA, USA) [196], and nanopore sequencing, using the MinION instrument (Oxford Nanopore Technologies, Oxford, UK) [197], will fill the gap of massive parallel sequencing, with long reads (over 10 kb), and alignment and mapping errors reduction. LRS improves the identification of structural variants, such as large inversions and translocations, and pseudogenes, as well as precisely sequence long tandem repeat expansions and high GC-rich regions, increases variant phasing determination, allowing the simultaneous establishment of parental origin, inheritance patterns, and disease risk haplotypes [198].

While the current variant classification guidelines combine functional and clinical data, the stepwise ABC system proposed by Houge et al. [199] suggests a sequential combination of the (A) functional and (B) clinical grades and optionally (C) selection of a standard comment(s) that best address the clinical question. In order to guide clinicians in attaining variant significance, the ABC system can be used separately or as an add-on the ACMG/AMP classification. This highlights the need and the importance of the crosstalk between clinicians and laboratory geneticists to guide genetic investigation, to establish (novel) genotype-phenotype correlations and ultimately to understand the mechanisms underlying the diseases.

The current challenge is the evaluation of the pathogenicity of the variants, rather than their identification. For this purpose, multidisciplinary international research collaborations/cooperation must be established. Ideally, a “rapid” functional test to study several genes in a diagnostic setting, might contribute to overcome this issue. This could represent an important step to translate these insights into future applications that will improve personalized patient support, care and treatment.

## Data Availability

Data sharing is not applicable to this article as no datasets were generated or analysed during the current study.

## Abbreviations

ACMG: American College of Medical Genetics
ADHD: Attention deficit hyperactivity disorder
ADID: Autosomal dominant intellectual disability
AMP: Association for Molecular Pathology
ASD: Autism spectrum disorder
APS: Adenosine 5′ phosphosulfate
ARID: Autosomal recessive intellectual disability
ATP: Adenosine triphosphate
BAM: Binary Alignment Map
BMP: Bone morphogenetic protein
CA: California
CADD: Combined Annotation Dependent Depletion
Cas9: CRISPR associated protein 9
*CLCN4*: Chloride channel 4 gene
CMA: Chromosomal microarray
*CNKSR2*: Connector enhancer of kinase suppressor of RAS 2 gene
CNS: Central nervous system
CNVs: Copy number variations
CRISPR: Clustered regularly interspaced short palindromic repeats
CRISPRa: CRISPR activation
CRISPRi: CRISPR interference
CSAS: Canonical splicing acceptor site
CSDS: Canonical splicing donor site
DBS: Double-strand breaks
dCas9: Dead Cas9
DD: Developmental delay
DDD: Deciphering Developmental Disorders
DNA: Deoxyribonucleic acid
DGV: Database of genomic variants
DSM-5: Diagnostic and Statistical Manual of Mental Disorders, 5th Edition
ES: Exome sequencing
FISH: Fluorescence in situ hybridization
*FMR1*: FMRP translational regulator 1 gene
*FRMPD4*: FERM and PDZ domains-containing protein 4 gene
FXS: Fragile X syndrome
GABA: Gamma aminobutyric acid
*GALT*: Galactose-1-phosphate uridylyltransferase gene
GDD: Global developmental delay
*GLI3*: Gli-Kruppel family member 3 gene
gRNA: Guide RNA
GS: Genome sequencing
HDR: Homology-directed repair
hiPSCs: Human induced pluripotent stem cells
ID: Intellectual disability
Indels: insertions or deletions
IQ: Intelligence quotient
*KLHL15*: Kelch-like 15 gene
*LAS1L*: LAS1-like ribosome biogenesis factor gene
LoF: Loss of function
LRS: Long-read sequencing
MAF: Minor allele frequency
*MECP2*: Methyl-CpG-binding protein 2 gene
MED12: Mediator complex subunit 12
MES: Medical exome sequencing
MT: Mitochondrial
MtID: Mitochondrial intellectual disability
mtDNA: Mitochondrial DNA
*NAA10*: N-alpha-acetyltransferase 10, NatA catalytic subunit gene
NDDs: Neurodevelopmental disorders
NGS: Next generation sequencing
NHEJ: Nonhomologous end joining
NSID: Non-syndromic intellectual disability
P1: Primer 1
*PAH*: Phenylalanine Hydroxylase gene
PPi: Inorganic pyrophosphate
*PTEN*: Phosphatase and tensin homolog gene
*RLIM*: Ring finger protein, lim domain-interacting gene
RNA: Ribonucleic acid
SAS: Splicing acceptor site
SDS: Splicing donor site
SID: Syndromic intellectual disability
SMRT: Single-molecule real-time
SNP: Single nucleotide polymorphism
SNVs: Single nucleotide variants
SVs: Structural variants
*SYNGAP1*: Synaptic RAS-GTPase-activating protein 1 gene
TNGS: Targeted-NGS
TOR: Target of rapamycin
UK: United Kingdom
USA: United States of America
*USP27X*: Ubiquitin-specific protease 27, X-linked gene
VAF: Varian frequency allele
VCF: Variant call format
VUS: Variant-of-unknown-significance
XLID: X-linked intellectual disability

## References

[1] Association AP. Diagnostic and statistical manual of mental disorders. 5th ed. Arlington: The American Psychiatric Association; 2013.

[2] de Ligt J, Willemsen MH, van Bon BW, Kleefstra T, Yntema HG, Kroes T, Vulto-van Silfhout AT, Koolen DA, de Vries P, Gilissen C, del Rosario M, Hoischen A, Scheffer H, de Vries BB, Brunner HG, Veltman JA, Vissers LE (2012). Diagnostic exome sequencing in persons with severe intellectual disability. N Engl J Med.

[3] Mitchell KJ. The genetic architecture of neurodevelopmental disorders. In: The genetics of neurodevelopmental disorders. Hoboken: Wiley; 2015. p. 1–28.

[4] Kochinke K, Zweier C, Nijhof B, Fenckova M, Cizek P, Honti F, Keerthikumar S, Oortveld MA, Kleefstra T, Kramer JM, Webber C, Huynen MA, Schenck A (2016). Systematic phenomics analysis deconvolutes genes mutated in intellectual disability into biologically coherent modules. Am J Hum Genet.

[5] Hu H, Haas SA, Chelly J, Van Esch H, Raynaud M, de Brouwer AP, Weinert S, Froyen G, Frints SG, Laumonnier F, Zemojtel T, Love MI, Richard H, Emde AK, Bienek M, Jensen C, Hambrock M, Fischer U, Langnick C, Feldkamp M, Wissink-Lindhout W, Lebrun N, Castelnau L, Rucci J, Montjean R, Dorseuil O, Billuart P, Stuhlmann T, Shaw M, Corbett MA, Gardner A, Willis-Owen S, Tan C, Friend KL, Belet S, van Roozendaal KE, Jimenez-Pocquet M, Moizard MP, Ronce N, Sun R, O'Keeffe S, Chenna R, van Bommel A, Goke J, Hackett A, Field M, Christie L, Boyle J, Haan E, Nelson J, Turner G, Baynam G, Gillessen-Kaesbach G, Muller U, Steinberger D, Budny B, Badura-Stronka M, Latos-Bielenska A, Ousager LB, Wieacker P, Rodriguez Criado G, Bondeson ML, Anneren G, Dufke A, Cohen M, Van Maldergem L, Vincent-Delorme C, Echenne B, Simon-Bouy B, Kleefstra T, Willemsen M, Fryns JP, Devriendt K, Ullmann R, Vingron M, Wrogemann K, Wienker TF, Tzschach A, van Bokhoven H, Gecz J, Jentsch TJ, Chen W, Ropers HH, Kalscheuer VM (2016). X-exome sequencing of 405 unresolved families identifies seven novel intellectual disability genes. Mol Psychiatry.

[6] Vissers LE, Gilissen C, Veltman JA (2016). Genetic studies in intellectual disability and related disorders. Nat Rev Genet.

[7] Chiurazzi P, Pirozzi F (2016). Advances in understanding - genetic basis of intellectual disability. F1000Research.

[8] Zigler E (1967). Familial mental retardation: a continuing dilemma. Science (New York, NY).

[9] Leonard H, Wen X (2002). The epidemiology of mental retardation: challenges and opportunities in the new millennium. Ment Retard Dev Disabil Res Rev.

[10] Association AP. Diagnostic and statistical manual of mental disorders. 4th ed. Washington, DC: The American Psychiatric Association; 1994.

[11] Organization WH (2004). ICD-10: international statistical classification of diseases and related health problems: tenth revision.

[12] van Bokhoven H (2011). Genetic and epigenetic networks in intellectual disabilities. Annu Rev Genet.

[13] Katz G, Lazcano-Ponce E (2008). Intellectual disability: definition, etiological factors, classification, diagnosis, treatment and prognosis. Salud Publica Mex.

[14] Vasudevan P, Suri M (2017). A clinical approach to developmental delay and intellectual disability. Clin Med (London).

[15] Choo YY, Agarwal P, How CH, Yeleswarapu SP (2019). Developmental delay: identification and management at primary care level. Singap Med J.

[16] Patel DR, Cabral MD, Ho A, Merrick J (2020). A clinical primer on intellectual disability. Transl Pediatr.

[17] Westerinen H, Kaski M, Virta L, Almqvist F, Iivanainen M (2007). Prevalence of intellectual disability: a comprehensive study based on national registers. J Intellect Disabil Res.

[18] Salvador-Carulla L, Symonds S (2016). Health services use and costs in people with intellectual disability: building a context knowledge base for evidence-informed policy. Curr Opin Psychiatry.

[19] Ropers HH (2010). Genetics of early onset cognitive impairment. Annu Rev Genomics Hum Genet.

[20] Reichenberg A, Cederlof M, McMillan A, Trzaskowski M, Kapra O, Fruchter E, Ginat K, Davidson M, Weiser M, Larsson H, Plomin R, Lichtenstein P (2016). Discontinuity in the genetic and environmental causes of the intellectual disability spectrum. Proc Natl Acad Sci U S A.

[21] Rauch A, Hoyer J, Guth S, Zweier C, Kraus C, Becker C, Zenker M, Huffmeier U, Thiel C, Ruschendorf F, Nurnberg P, Reis A, Trautmann U (2006). Diagnostic yield of various genetic approaches in patients with unexplained developmental delay or mental retardation. Am J Med Genet A.

[22] Lejeune J, Turpin R, Gautier M (1959). Chromosomic diagnosis of mongolism. Arch Fr Pediatr.

[23] Sherman SL, Allen EG, Bean LH, Freeman SB (2007). Epidemiology of Down syndrome. Ment Retard Dev Disabil Res Rev.

[24] Michelson DJ, Shevell MI, Sherr EH, Moeschler JB, Gropman AL, Ashwal S (2011). Evidence report: genetic and metabolic testing on children with global developmental delay: report of the Quality Standards Subcommittee of the American Academy of Neurology and the Practice Committee of the Child Neurology Society. Neurology.

[25] Genetic A, The New York-Mid-Atlantic Consortium for G, Newborn Screening S (2009). Genetic Alliance monographs and guides. Understanding genetics: a New York, mid-Atlantic guide for patients and health professionals.

[26] Vissers LE, de Vries BB, Osoegawa K, Janssen IM, Feuth T, Choy CO, Straatman H, van der Vliet W, Huys EH, van Rijk A, Smeets D, van Ravenswaaij-Arts CM, Knoers NV, van der Burgt I, de Jong PJ, Brunner HG, van Kessel AG, Schoenmakers EF, Veltman JA (2003). Array-based comparative genomic hybridization for the genomewide detection of submicroscopic chromosomal abnormalities. Am J Hum Genet.

[27] Rauch A, Wieczorek D, Graf E, Wieland T, Endele S, Schwarzmayr T, Albrecht B, Bartholdi D, Beygo J, Di Donato N, Dufke A, Cremer K, Hempel M, Horn D, Hoyer J, Joset P, Ropke A, Moog U, Riess A, Thiel CT, Tzschach A, Wiesener A, Wohlleber E, Zweier C, Ekici AB, Zink AM, Rump A, Meisinger C, Grallert H, Sticht H, Schenck A, Engels H, Rappold G, Schrock E, Wieacker P, Riess O, Meitinger T, Reis A, Strom TM (2012). Range of genetic mutations associated with severe non-syndromic sporadic intellectual disability: an exome sequencing study. Lancet.

[28] Shaw-Smith C, Redon R, Rickman L, Rio M, Willatt L, Fiegler H, Firth H, Sanlaville D, Winter R, Colleaux L, Bobrow M, Carter NP (2004). Microarray based comparative genomic hybridisation (array-CGH) detects submicroscopic chromosomal deletions and duplications in patients with learning disability/mental retardation and dysmorphic features. J Med Genet.

[29] Wagenstaller J, Spranger S, Lorenz-Depiereux B, Kazmierczak B, Nathrath M, Wahl D, Heye B, Glaser D, Liebscher V, Meitinger T, Strom TM (2007). Copy-number variations measured by single-nucleotide-polymorphism oligonucleotide arrays in patients with mental retardation. Am J Hum Genet.

[30] Cooper GM, Coe BP, Girirajan S, Rosenfeld JA, Vu TH, Baker C, Williams C, Stalker H, Hamid R, Hannig V, Abdel-Hamid H, Bader P, McCracken E, Niyazov D, Leppig K, Thiese H, Hummel M, Alexander N, Gorski J, Kussmann J, Shashi V, Johnson K, Rehder C, Ballif BC, Shaffer LG, Eichler EE (2011). A copy number variation morbidity map of developmental delay. Nat Genet.

[31] Lupski JR, Stankiewicz P (2005). Genomic disorders: molecular mechanisms for rearrangements and conveyed phenotypes. PLoS Genet.

[32] Sanger F, Coulson AR (1975). A rapid method for determining sequences in DNA by primed synthesis with DNA polymerase. J Mol Biol.

[33] Smith LM, Sanders JZ, Kaiser RJ, Hughes P, Dodd C, Connell CR, Heiner C, Kent SB, Hood LE (1986). Fluorescence detection in automated DNA sequence analysis. Nature.

[34] Ansorge W, Sproat B, Stegemann J, Schwager C, Zenke M (1987). Automated DNA sequencing: ultrasensitive detection of fluorescent bands during electrophoresis. Nucleic Acids Res.

[35] Ansorge W, Sproat BS, Stegemann J, Schwager C (1986). A non-radioactive automated method for DNA sequence determination. J Biochem Biophys Methods.

[36] Verkerk AJ, Pieretti M, Sutcliffe JS, Fu YH, Kuhl DP, Pizzuti A, Reiner O, Richards S, Victoria MF, Zhang FP (1991). Identification of a gene (FMR-1) containing a CGG repeat coincident with a breakpoint cluster region exhibiting length variation in fragile X syndrome. Cell.

[37] Ciaccio C, Fontana L, Milani D, Tabano S, Miozzo M, Esposito S (2017). Fragile X syndrome: a review of clinical and molecular diagnoses. Ital J Pediatr.

[38] Paulson H (2019). Repeat expansions in leukoencephalopathy. Ann Neurol.

[39] Liquori CL, Ricker K, Moseley ML, Jacobsen JF, Kress W, Naylor SL, Day JW, Ranum LP (2001). Myotonic dystrophy type 2 caused by a CCTG expansion in intron 1 of ZNF9. Science (New York, NY).

[40] Matsuura T, Yamagata T, Burgess DL, Rasmussen A, Grewal RP, Watase K, Khajavi M, McCall AE, Davis CF, Zu L, Achari M, Pulst SM, Alonso E, Noebels JL, Nelson DL, Zoghbi HY, Ashizawa T (2000). Large expansion of the ATTCT pentanucleotide repeat in spinocerebellar ataxia type 10. Nat Genet.

[41] Seixas AI, Loureiro JR, Costa C, Ordonez-Ugalde A, Marcelino H, Oliveira CL, Loureiro JL, Dhingra A, Brandao E, Cruz VT, Timoteo A, Quintans B, Rouleau GA, Rizzu P, Carracedo A, Bessa J, Heutink P, Sequeiros J, Sobrido MJ, Coutinho P, Silveira I (2017). A pentanucleotide ATTTC repeat insertion in the non-coding region of DAB1, mapping to SCA37, causes spinocerebellar ataxia. Am J Hum Genet.

[42] Gatchel JR, Zoghbi HY (2005). Diseases of unstable repeat expansion: mechanisms and common principles. Nat Rev Genet.

[43] Jin Z, Liu Y (2018). DNA methylation in human diseases. Genes Dis.

[44] Reik W, Walter J (2001). Genomic imprinting: parental influence on the genome. Nat Rev Genet.

[45] Barbosa M, Joshi RS, Garg P, Martin-Trujillo A, Patel N, Jadhav B, Watson CT, Gibson W, Chetnik K, Tessereau C, Mei H, De Rubeis S, Reichert J, Lopes F, Vissers L, Kleefstra T, Grice DE, Edelmann L, Soares G, Maciel P, Brunner HG, Buxbaum JD, Gelb BD, Sharp AJ (2018). Identification of rare de novo epigenetic variations in congenital disorders. Nat Commun.

[46] Hannon E, Gorrie-Stone TJ, Smart MC, Burrage J, Hughes A, Bao Y, Kumari M, Schalkwyk LC, Mill J (2018). Leveraging DNA-methylation quantitative-trait loci to characterize the relationship between methylomic variation, gene expression, and complex traits. Am J Hum Genet.

[47] Peters J (2014). The role of genomic imprinting in biology and disease: an expanding view. Nat Rev Genet.

[48] Cassidy SB, Schwartz S, Miller JL, Driscoll DJ (2012). Prader-Willi syndrome. Genet Med.

[49] Williams CA, Driscoll DJ, Dagli AI (2010). Clinical and genetic aspects of Angelman syndrome. Genet Med.

[50] Wakeling EL, Amero SA, Alders M, Bliek J, Forsythe E, Kumar S, Lim DH, MacDonald F, Mackay DJ, Maher ER, Moore GE, Poole RL, Price SM, Tangeraas T, Turner CL, Van Haelst MM, Willoughby C, Temple IK, Cobben JM (2010). Epigenotype-phenotype correlations in Silver-Russell syndrome. J Med Genet.

[51] Weksberg R, Shuman C, Beckwith JB (2010). Beckwith-Wiedemann syndrome. Eur J Hum Genet.

[52] Ioannides Y, Lokulo-Sodipe K, Mackay DJ, Davies JH, Temple IK (2014). Temple syndrome: improving the recognition of an underdiagnosed chromosome 14 imprinting disorder: an analysis of 51 published cases. J Med Genet.

[53] Kagami M, Sekita Y, Nishimura G, Irie M, Kato F, Okada M, Yamamori S, Kishimoto H, Nakayama M, Tanaka Y, Matsuoka K, Takahashi T, Noguchi M, Tanaka Y, Masumoto K, Utsunomiya T, Kouzan H, Komatsu Y, Ohashi H, Kurosawa K, Kosaki K, Ferguson-Smith AC, Ishino F, Ogata T (2008). Deletions and epimutations affecting the human 14q32.2 imprinted region in individuals with paternal and maternal upd(14)-like phenotypes. Nat Genet.

[54] Docherty LE, Kabwama S, Lehmann A, Hawke E, Harrison L, Flanagan SE, Ellard S, Hattersley AT, Shield JP, Ennis S, Mackay DJ, Temple IK (2013). Clinical presentation of 6q24 transient neonatal diabetes mellitus (6q24 TNDM) and genotype-phenotype correlation in an international cohort of patients. Diabetologia.

[55] Bastepe M (2008). The GNAS locus and pseudohypoparathyroidism. Adv Exp Med Biol.

[56] Craven L, Alston CL, Taylor RW, Turnbull DM (2017). Recent advances in mitochondrial disease. Annu Rev Genomics Hum Genet.

[57] Gorman GS, Schaefer AM, Ng Y, Gomez N, Blakely EL, Alston CL, Feeney C, Horvath R, Yu-Wai-Man P, Chinnery PF, Taylor RW, Turnbull DM, McFarland R (2015). Prevalence of nuclear and mitochondrial DNA mutations related to adult mitochondrial disease. Ann Neurol.

[58] Elliott HR, Samuels DC, Eden JA, Relton CL, Chinnery PF (2008). Pathogenic mitochondrial DNA mutations are common in the general population. Am J Hum Genet.

[59] Lake NJ, Compton AG, Rahman S, Thorburn DR (2016). Leigh syndrome: one disorder, more than 75 monogenic causes. Ann Neurol.

[60] Naviaux RK, Nguyen KV (2004). POLG mutations associated with Alpers’ syndrome and mitochondrial DNA depletion. Ann Neurol.

[61] Chelly J, Khelfaoui M, Francis F, Cherif B, Bienvenu T (2006). Genetics and pathophysiology of mental retardation. Eur J Hum Genet.

[62] Sanchez-Mut JV, Huertas D, Esteller M (2012). Aberrant epigenetic landscape in intellectual disability. Prog Brain Res.

[63] Darvish H, Azcona LJ, Tafakhori A, Mesias R, Ahmadifard A, Sanchez E, Habibi A, Alehabib E, Johari AH, Emamalizadeh B, Jamali F, Chapi M, Jamshidi J, Kajiwara Y, Paisan-Ruiz C (2020). Phenotypic and genotypic characterization of families with complex intellectual disability identified pathogenic genetic variations in known and novel disease genes. Sci Rep.

[64] Roca I, Fernandez-Marmiesse A, Gouveia S, Segovia M, Couce ML (2018). Prioritization of variants detected by next generation sequencing according to the mutation tolerance and mutational architecture of the corresponding genes. Int J Mol Sci.

[65] Bruel AL, Vitobello A, Tran Mau-Them F, Nambot S, Sorlin A, Denomme-Pichon AS, Delanne J, Moutton S, Callier P, Duffourd Y, Philippe C, Faivre L, Thauvin-Robinet C (2020). Next-generation sequencing approaches and challenges in the diagnosis of developmental anomalies and intellectual disability. Clin Genet.

[66] Ng SB, Buckingham KJ, Lee C, Bigham AW, Tabor HK, Dent KM, Huff CD, Shannon PT, Jabs EW, Nickerson DA, Shendure J, Bamshad MJ (2010). Exome sequencing identifies the cause of a mendelian disorder. Nat Genet.

[67] Gilissen C, Hehir-Kwa JY, Thung DT, van de Vorst M, van Bon BW, Willemsen MH, Kwint M, Janssen IM, Hoischen A, Schenck A, Leach R, Klein R, Tearle R, Bo T, Pfundt R, Yntema HG, de Vries BB, Kleefstra T, Brunner HG, Vissers LE, Veltman JA (2014). Genome sequencing identifies major causes of severe intellectual disability. Nature.

[68] Jamra R (2018). Genetics of autosomal recessive intellectual disability. Med Genet.

[69] Garcia-Cazorla A, Wolf NI, Serrano M, Moog U, Perez-Duenas B, Poo P, Pineda M, Campistol J, Hoffmann GF (2009). Mental retardation and inborn errors of metabolism. J Inherit Metab Dis.

[70] Muzzey D, Evans EA, Lieber C (2015). Understanding the basics of NGS: from mechanism to variant calling. Curr Genet Med Rep.

[71] Metzker ML (2010). Sequencing technologies - the next generation. Nat Rev Genet.

[72] Ju J, Kim DH, Bi L, Meng Q, Bai X, Li Z, Li X, Marma MS, Shi S, Wu J, Edwards JR, Romu A, Turro NJ (2006). Four-color DNA sequencing by synthesis using cleavable fluorescent nucleotide reversible terminators. Proc Natl Acad Sci U S A.

[73] Harrington CT, Lin EI, Olson MT, Eshleman JR (2013). Fundamentals of pyrosequencing. Arch Pathol Lab Med.

[74] Porreca GJ, Shendure J, Church GM (2006). Polony DNA sequencing. Curr Protoc Mol Biol.

[75] Budczies J, Bockmayr M, Treue D, Klauschen F, Denkert C (2015). Semiconductor sequencing: how many flows do you need?. Bioinformatics (Oxford, England).

[76] Najmabadi H, Hu H, Garshasbi M, Zemojtel T, Abedini SS, Chen W, Hosseini M, Behjati F, Haas S, Jamali P, Zecha A, Mohseni M, Puttmann L, Vahid LN, Jensen C, Moheb LA, Bienek M, Larti F, Mueller I, Weissmann R, Darvish H, Wrogemann K, Hadavi V, Lipkowitz B, Esmaeeli-Nieh S, Wieczorek D, Kariminejad R, Firouzabadi SG, Cohen M, Fattahi Z, Rost I, Mojahedi F, Hertzberg C, Dehghan A, Rajab A, Banavandi MJ, Hoffer J, Falah M, Musante L, Kalscheuer V, Ullmann R, Kuss AW, Tzschach A, Kahrizi K, Ropers HH (2011). Deep sequencing reveals 50 novel genes for recessive cognitive disorders. Nature.

[77] Tzschach A, Grasshoff U, Beck-Woedl S, Dufke C, Bauer C, Kehrer M, Evers C, Moog U, Oehl-Jaschkowitz B, Di Donato N, Maiwald R, Jung C, Kuechler A, Schulz S, Meinecke P, Spranger S, Kohlhase J, Seidel J, Reif S, Rieger M, Riess A, Sturm M, Bickmann J, Schroeder C, Dufke A, Riess O, Bauer P (2015). Next-generation sequencing in X-linked intellectual disability. Eur J Hum Genet.

[78] Hu H, Kahrizi K, Musante L, Fattahi Z, Herwig R, Hosseini M, Oppitz C, Abedini SS, Suckow V, Larti F, Beheshtian M, Lipkowitz B, Akhtarkhavari T, Mehvari S, Otto S, Mohseni M, Arzhangi S, Jamali P, Mojahedi F, Taghdiri M, Papari E, Soltani Banavandi MJ, Akbari S, Tonekaboni SH, Dehghani H, Ebrahimpour MR, Bader I, Davarnia B, Cohen M, Khodaei H, Albrecht B, Azimi S, Zirn B, Bastami M, Wieczorek D, Bahrami G, Keleman K, Vahid LN, Tzschach A, Gartner J, Gillessen-Kaesbach G, Varaghchi JR, Timmermann B, Pourfatemi F, Jankhah A, Chen W, Nikuei P, Kalscheuer VM, Oladnabi M, Wienker TF, Ropers HH, Najmabadi H (2019). Genetics of intellectual disability in consanguineous families. Mol Psychiatry.

[79] Mir YR, Kuchay RAH (2019). Advances in identification of genes involved in autosomal recessive intellectual disability: a brief review. J Med Genet.

[80] Harripaul R, Noor A, Ayub M, Vincent JB (2017). The use of next-generation sequencing for research and diagnostics for intellectual disability. Cold Spring Harb Perspect Med.

[81] Rehm HL (2013). Disease-targeted sequencing: a cornerstone in the clinic. Nat Rev Genet.

[82] Malaga DR, Brusius-Facchin AC, Siebert M, Pasqualim G, Saraiva-Pereira ML, Souza CFM, Schwartz IVD, Matte U, Giugliani R (2019). Sensitivity, advantages, limitations, and clinical utility of targeted next-generation sequencing panels for the diagnosis of selected lysosomal storage disorders. Genet Mol Biol.

[83] Gordon LG, White NM, Elliott TM, Nones K, Beckhouse AG, Rodriguez-Acevedo AJ, Webb PM, Lee XJ, Graves N, Schofield DJ (2020). Estimating the costs of genomic sequencing in cancer control. BMC Health Serv Res.

[84] Warr A, Robert C, Hume D, Archibald A, Deeb N, Watson M (2015). Exome sequencing: current and future perspectives. G3 (Bethesda).

[85] Wright CF, Fitzgerald TW, Jones WD, Clayton S, McRae JF, van Kogelenberg M, King DA, Ambridge K, Barrett DM, Bayzetinova T, Bevan AP, Bragin E, Chatzimichali EA, Gribble S, Jones P, Krishnappa N, Mason LE, Miller R, Morley KI, Parthiban V, Prigmore E, Rajan D, Sifrim A, Swaminathan GJ, Tivey AR, Middleton A, Parker M, Carter NP, Barrett JC, Hurles ME, FitzPatrick DR, Firth HV (2015). Genetic diagnosis of developmental disorders in the DDD study: a scalable analysis of genome-wide research data. Lancet.

[86] Wright CF, JF MR, Clayton S, Gallone G, Aitken S, FitzGerald TW, Jones P, Prigmore E, Rajan D, Lord J, Sifrim A, Kelsell R, Parker MJ, Barrett JC, Hurles ME, FitzPatrick DR, Firth HV, Study DDD (2018). Making new genetic diagnoses with old data: iterative reanalysis and reporting from genome-wide data in 1,133 families with developmental disorders. Genet Med.

[87] Srivastava S, Love-Nichols JA, Dies KA, Ledbetter DH, Martin CL, Chung WK, Firth HV, Frazier T, Hansen RL, Prock L, Brunner H, Hoang N, Scherer SW, Sahin M, Miller DT, Group NDDESRW (2019). Meta-analysis and multidisciplinary consensus statement: exome sequencing is a first-tier clinical diagnostic test for individuals with neurodevelopmental disorders. Genet Med.

[88] Minoche AE, Lundie B, Peters GB, Ohnesorg T, Pinese M, Thomas DM, Zankl A, Roscioli T, Schonrock N, Kummerfeld S, Burnett L, Dinger ME, Cowley MJ (2021). ClinSV: clinical grade structural and copy number variant detection from whole genome sequencing data. Genome Med.

[89] Cao Y, Tokita MJ, Chen ES, Ghosh R, Chen T, Feng Y, Gorman E, Gibellini F, Ward PA, Braxton A, Wang X, Meng L, Xiao R, Bi W, Xia F, Eng CM, Yang Y, Gambin T, Shaw C, Liu P, Stankiewicz P (2019). A clinical survey of mosaic single nucleotide variants in disease-causing genes detected by exome sequencing. Genome Med.

[90] Schluth-Bolard C, Labalme A, Cordier MP, Till M, Nadeau G, Tevissen H, Lesca G, Boutry-Kryza N, Rossignol S, Rocas D, Dubruc E, Edery P, Sanlaville D (2013). Breakpoint mapping by next generation sequencing reveals causative gene disruption in patients carrying apparently balanced chromosome rearrangements with intellectual deficiency and/or congenital malformations. J Med Genet.

[91] Bowling KM, Thompson ML, Amaral MD, Finnila CR, Hiatt SM, Engel KL, Cochran JN, Brothers KB, East KM, Gray DE, Kelley WV, Lamb NE, Lose EJ, Rich CA, Simmons S, Whittle JS, Weaver BT, Nesmith AS, Myers RM, Barsh GS, Bebin EM, Cooper GM (2017). Genomic diagnosis for children with intellectual disability and/or developmental delay. Genome Med.

[92] Wang J, Wang Y, Wang L, Chen WY, Sheng M (2020). The diagnostic yield of intellectual disability: combined whole genome low-coverage sequencing and medical exome sequencing. BMC Med Genet.

[93] Pua CJ, Bhalshankar J, Miao K, Walsh R, John S, Lim SQ, Chow K, Buchan R, Soh BY, Lio PM, Lim J, Schafer S, Lim JQ, Tan P, Whiffin N, Barton PJ, Ware JS, Cook SA (2016). Development of a comprehensive sequencing assay for inherited cardiac condition genes. J Cardiovasc Transl Res.

[94] Cock PJ, Fields CJ, Goto N, Heuer ML, Rice PM (2010). The Sanger FASTQ file format for sequences with quality scores, and the Solexa/Illumina FASTQ variants. Nucleic Acids Res.

[95] Li H, Handsaker B, Wysoker A, Fennell T, Ruan J, Homer N, Marth G, Abecasis G, Durbin R, Genome Project Data Processing S (2009). The sequence alignment/map format and SAMtools. Bioinformatics (Oxford, England).

[96] Danecek P, Auton A, Abecasis G, Albers CA, Banks E, DePristo MA, Handsaker RE, Lunter G, Marth GT, Sherry ST, McVean G, Durbin R, Genomes Project Analysis G (2011). The variant call format and VCFtools. Bioinformatics (Oxford, England).

[97] Koboldt DC (2020). Best practices for variant calling in clinical sequencing. Genome Med.

[98] Rohlin A, Wernersson J, Engwall Y, Wiklund L, Bjork J, Nordling M (2009). Parallel sequencing used in detection of mosaic mutations: comparison with four diagnostic DNA screening techniques. Hum Mutat.

[99] Jamuar SS, Lam AT, Kircher M, D'Gama AM, Wang J, Barry BJ, Zhang X, Hill RS, Partlow JN, Rozzo A, Servattalab S, Mehta BK, Topcu M, Amrom D, Andermann E, Dan B, Parrini E, Guerrini R, Scheffer IE, Berkovic SF, Leventer RJ, Shen Y, Wu BL, Barkovich AJ, Sahin M, Chang BS, Bamshad M, Nickerson DA, Shendure J, Poduri A, Yu TW, Walsh CA (2014). Somatic mutations in cerebral cortical malformations. N Engl J Med.

[100] Howe KL, Achuthan P, Allen J, Allen J, Alvarez-Jarreta J, Amode MR, Armean IM, Azov AG, Bennett R, Bhai J, Billis K, Boddu S, Charkhchi M, Cummins C, Da Rin Fioretto L, Davidson C, Dodiya K, El Houdaigui B, Fatima R, Gall A, Garcia Giron C, Grego T, Guijarro-Clarke C, Haggerty L, Hemrom A, Hourlier T, Izuogu OG, Juettemann T, Kaikala V, Kay M, Lavidas I, Le T, Lemos D, Gonzalez Martinez J, Marugan JC, Maurel T, McMahon AC, Mohanan S, Moore B, Muffato M, Oheh DN, Paraschas D, Parker A, Parton A, Prosovetskaia I, Sakthivel MP, Salam AIA, Schmitt BM, Schuilenburg H, Sheppard D, Steed E, Szpak M, Szuba M, Taylor K, Thormann A, Threadgold G, Walts B, Winterbottom A, Chakiachvili M, Chaubal A, De Silva N, Flint B, Frankish A, Hunt SE GR, Langridge N, Loveland JE, Martin FJ, Mudge JM, Morales J, Perry E, Ruffier M, Tate J, Thybert D, Trevanion SJ, Cunningham F, Yates AD, Zerbino DR, Flicek P (2021). Ensembl 2021. Nucleic Acids Res.

[101] Sherry ST, Ward MH, Kholodov M, Baker J, Phan L, Smigielski EM, Sirotkin K (2001). dbSNP: the NCBI database of genetic variation. Nucleic Acids Res.

[102] Karczewski KJ, Francioli LC, Tiao G, Cummings BB, Alfoldi J, Wang Q, Collins RL, Laricchia KM, Ganna A, Birnbaum DP, Gauthier LD, Brand H, Solomonson M, Watts NA, Rhodes D, Singer-Berk M, England EM, Seaby EG, Kosmicki JA, Walters RK, Tashman K, Farjoun Y, Banks E, Poterba T, Wang A, Seed C, Whiffin N, Chong JX, Samocha KE, Pierce-Hoffman E, Zappala Z, O'Donnell-Luria AH, Minikel EV, Weisburd B, Lek M, Ware JS, Vittal C, Armean IM, Bergelson L, Cibulskis K, Connolly KM, Covarrubias M, Donnelly S, Ferriera S, Gabriel S, Gentry J, Gupta N, Jeandet T, Kaplan D, Llanwarne C, Munshi R, Novod S, Petrillo N, Roazen D, Ruano-Rubio V, Saltzman A, Schleicher M, Soto J, Tibbetts K, Tolonen C, Wade G, Talkowski ME, Neale BM, Daly MJ, MacArthur DG, Genome Aggregation Database C (2020). The mutational constraint spectrum quantified from variation in 141,456 humans. Nature.

[103] MacDonald JR, Ziman R, Yuen RK, Feuk L, Scherer SW (2014). The database of genomic variants: a curated collection of structural variation in the human genome. Nucleic Acids Res.

[104] Firth HV, Richards SM, Bevan AP, Clayton S, Corpas M, Rajan D, Van Vooren S, Moreau Y, Pettett RM, Carter NP (2009). DECIPHER: database of chromosomal imbalance and phenotype in humans Using Ensembl resources. Am J Hum Genet.

[105] Papadimitriou S, Gazzo A, Versbraegen N, Nachtegael C, Aerts J, Moreau Y, Van Dooren S, Nowe A, Smits G, Lenaerts T (2019). Predicting disease-causing variant combinations. Proc Natl Acad Sci U S A.

[106] Mani A (2017). Pathogenicity of de novo rare variants: challenges and opportunities. Circ Cardiovasc Genet.

[107] Niemi MEK, Martin HC, Rice DL, Gallone G, Gordon S, Kelemen M, McAloney K, McRae J, Radford EJ, Yu S, Gecz J, Martin NG, Wright CF, Fitzpatrick DR, Firth HV, Hurles ME, Barrett JC (2018). Common genetic variants contribute to risk of rare severe neurodevelopmental disorders. Nature.

[108] Consortium EP (2004). The ENCODE (ENCyclopedia Of DNA Elements) Project. Science (New York, NY).

[109] Gussow AB, Copeland BR, Dhindsa RS, Wang Q, Petrovski S, Majoros WH, Allen AS, Goldstein DB (2017). Orion: detecting regions of the human non-coding genome that are intolerant to variation using population genetics. PLoS One.

[110] MacArthur DG, Manolio TA, Dimmock DP, Rehm HL, Shendure J, Abecasis GR, Adams DR, Altman RB, Antonarakis SE, Ashley EA, Barrett JC, Biesecker LG, Conrad DF, Cooper GM, Cox NJ, Daly MJ, Gerstein MB, Goldstein DB, Hirschhorn JN, Leal SM, Pennacchio LA, Stamatoyannopoulos JA, Sunyaev SR, Valle D, Voight BF, Winckler W, Gunter C (2014). Guidelines for investigating causality of sequence variants in human disease. Nature.

[111] Piton A, Redin C, Mandel JL (2013). XLID-causing mutations and associated genes challenged in light of data from large-scale human exome sequencing. Am J Hum Genet.

[112] Robinson JT, Thorvaldsdottir H, Winckler W, Guttman M, Lander ES, Getz G, Mesirov JP (2011). Integrative genomics viewer. Nat Biotechnol.

[113] Arteche-Lopez A, Avila-Fernandez A, Romero R, Riveiro-Alvarez R, Lopez-Martinez MA, Gimenez-Pardo A, Velez-Monsalve C, Gallego-Merlo J, Garcia-Vara I, Almoguera B, Bustamante-Aragones A, Blanco-Kelly F, Tahsin-Swafiri S, Rodriguez-Pinilla E, Minguez P, Lorda I, Trujillo-Tiebas MJ, Ayuso C (2021). Sanger sequencing is no longer always necessary based on a single-center validation of 1109 NGS variants in 825 clinical exomes. Sci Rep.

[114] Rehm HL, Bale SJ, Bayrak-Toydemir P, Berg JS, Brown KK, Deignan JL, Friez MJ, Funke BH, Hegde MR, Lyon E, Working Group of the American College of Medical G, Genomics Laboratory Quality Assurance C (2013). ACMG clinical laboratory standards for next-generation sequencing. Genet Med.

[115] Richards S, Aziz N, Bale S, Bick D, Das S, Gastier-Foster J, Grody WW, Hegde M, Lyon E, Spector E, Voelkerding K, Rehm HL (2015). Standards and guidelines for the interpretation of sequence variants: a joint consensus recommendation of the American College of Medical Genetics and Genomics and the Association for Molecular Pathology. Genet Med.

[116] Schuurs-Hoeijmakers JH, Vulto-van Silfhout AT, Vissers LE, van de Vondevoort IIGM, van Bon BW, de Ligt J, Gilissen C, Hehir-Kwa JY, Neveling K, del Rosario M, Hira G, Reitano S, Vitello A, Failla P, Greco D, Fichera M, Galesi O, Kleefstra T, Greally MT, Ockeloen CW, Willemsen MH, Bongers EM, Janssen IM, Pfundt R, Veltman JA, Romano C, Willemsen MA, van Bokhoven H, Brunner HG, de Vries BB, de Brouwer AP (2013). Identification of pathogenic gene variants in small families with intellectually disabled siblings by exome sequencing. J Med Genet.

[117] Landrum MJ, Lee JM, Benson M, Brown GR, Chao C, Chitipiralla S, Gu B, Hart J, Hoffman D, Jang W, Karapetyan K, Katz K, Liu C, Maddipatla Z, Malheiro A, McDaniel K, Ovetsky M, Riley G, Zhou G, Holmes JB, Kattman BL, Maglott DR (2018). ClinVar: improving access to variant interpretations and supporting evidence. Nucleic Acids Res.

[118] Rehm HL, Berg JS, Brooks LD, Bustamante CD, Evans JP, Landrum MJ, Ledbetter DH, Maglott DR, Martin CL, Nussbaum RL, Plon SE, Ramos EM, Sherry ST, Watson MS, ClinGen (2015). ClinGen--the clinical genome resource. N Engl J Med.

[119] Hamosh A, Scott AF, Amberger JS, Bocchini CA, McKusick VA (2005). Online Mendelian Inheritance in Man (OMIM), a knowledgebase of human genes and genetic disorders. Nucleic Acids Res.

[120] Amberger JS, Bocchini CA, Scott AF, Hamosh A (2019). OMIM.org: leveraging knowledge across phenotype-gene relationships. Nucleic Acids Res.

[121] Kohler S, Vasilevsky NA, Engelstad M, Foster E, McMurry J, Ayme S, Baynam G, Bello SM, Boerkoel CF, Boycott KM, Brudno M, Buske OJ, Chinnery PF, Cipriani V, Connell LE, Dawkins HJ, DeMare LE, Devereau AD, de Vries BB, Firth HV, Freson K, Greene D, Hamosh A, Helbig I, Hum C, Jahn JA, James R, Krause R, Laulederkind SJF, Lochmuller H, Lyon GJ, Ogishima S, Olry A, Ouwehand WH, Pontikos N, Rath A, Schaefer F, Scott RH, Segal M, Sergouniotis PI, Sever R, Smith CL, Straub V, Thompson R, Turner C, Turro E, Veltman MW, Vulliamy T, Yu J, von Ziegenweidt J, Zankl A, Zuchner S, Zemojtel T, Jacobsen JO, Groza T, Smedley D, Mungall CJ, Haendel M, Robinson PN (2017). The human phenotype ontology in 2017. Nucleic Acids Res.

[122] Papatheodorou I, Fonseca NA, Keays M, Tang YA, Barrera E, Bazant W, Burke M, Fullgrabe A, Fuentes AM, George N, Huerta L, Koskinen S, Mohammed S, Geniza M, Preece J, Jaiswal P, Jarnuczak AF, Huber W, Stegle O, Vizcaino JA, Brazma A, Petryszak R (2018). Expression atlas: gene and protein expression across multiple studies and organisms. Nucleic Acids Res.

[123] Carithers LJ, Ardlie K, Barcus M, Branton PA, Britton A, Buia SA, Compton CC, DS DL, Peter-Demchok J, Gelfand ET, Guan P, Korzeniewski GE, Lockhart NC, Rabiner CA, Rao AK, Robinson KL, Roche NV, Sawyer SJ, Segre AV, Shive CE, Smith AM, Sobin LH, Undale AH, Valentino KM, Vaught J, Young TR, Moore HM, Consortium GT (2015). A novel approach to high-quality postmortem tissue procurement: the GTEx project. Biopreserv Biobank.

[124] UniProt C (2019). UniProt: a worldwide hub of protein knowledge. Nucleic Acids Res.

[125] Hermjakob H, Montecchi-Palazzi L, Lewington C, Mudali S, Kerrien S, Orchard S, Vingron M, Roechert B, Roepstorff P, Valencia A, Margalit H, Armstrong J, Bairoch A, Cesareni G, Sherman D, Apweiler R (2004). IntAct: an open source molecular interaction database. Nucleic Acids Res.

[126] Stelzer G, Rosen N, Plaschkes I, Zimmerman S, Twik M, Fishilevich S, Stein TI, Nudel R, Lieder I, Mazor Y, Kaplan S, Dahary D, Warshawsky D, Guan-Golan Y, Kohn A, Rappaport N, Safran M, Lancet D (2016). The GeneCards Suite: from gene data mining to disease genome sequence analyses. Curr Protoc Bioinformatics.

[127] Shapiro MB, Senapathy P (1987). RNA splice junctions of different classes of eukaryotes: sequence statistics and functional implications in gene expression. Nucleic Acids Res.

[128] Yeo G, Burge CB (2004). Maximum entropy modeling of short sequence motifs with applications to RNA splicing signals. J Comput Biol.

[129] Reese MG, Eeckman FH, Kulp D, Haussler D (1997). Improved splice site detection in Genie. J Comput Biol.

[130] Pertea M, Lin X, Salzberg SL (2001). GeneSplicer: a new computational method for splice site prediction. Nucleic Acids Res.

[131] Kircher M, Witten DM, Jain P, O'Roak BJ, Cooper GM (2014). A general framework for estimating the relative pathogenicity of human genetic variants. Nat Genet.

[132] Au PYB, You J, Caluseriu O, Schwartzentruber J, Majewski J, Bernier FP, Ferguson M, Valle D, Parboosingh JS, Sobreira N, Innes AM, Kline AD, Care for Rare Canada C (2015). GeneMatcher aids in the identification of a new malformation syndrome with intellectual disability, unique facial dysmorphisms, and skeletal and connective tissue abnormalities caused by de novo variants in HNRNPK. Hum Mutat.

[133] Sobreira N, Schiettecatte F, Valle D, Hamosh A (2015). GeneMatcher: a matching tool for connecting investigators with an interest in the same gene. Hum Mutat.

[134] Dingemans AJM, Stremmelaar DE, Vissers L, Jansen S, Nabais Sa MJ, van Remortele A, Jonis N, Truijen K, van de Ven S, Ewals J, Verbruggen M, Koolen DA, Brunner HG, Eichler EE, Gecz J, de Vries BBA (2021). Human disease genes website series: an international, open and dynamic library for up-to-date clinical information. Am J Med Genet A.

[135] Buske OJ, Girdea M, Dumitriu S, Gallinger B, Hartley T, Trang H, Misyura A, Friedman T, Beaulieu C, Bone WP, Links AE, Washington NL, Haendel MA, Robinson PN, Boerkoel CF, Adams D, Gahl WA, Boycott KM, Brudno M (2015). PhenomeCentral: a portal for phenotypic and genotypic matchmaking of patients with rare genetic diseases. Hum Mutat.

[136] Fokkema IF, Taschner PE, Schaafsma GC, Celli J, Laros JF, den Dunnen JT (2011). LOVD v.2.0: the next generation in gene variant databases. Hum Mutat.

[137] Zurek B, Ellwanger K, Vissers L, Schule R, Synofzik M, Topf A, de Voer RM, Laurie S, Matalonga L, Gilissen C, Ossowski S, t Hoen PAC, Vitobello A, Schulze-Hentrich JM, Riess O, Brunner HG, Brookes AJ, Rath A, Bonne G, Gumus G, Verloes A, Hoogerbrugge N, Evangelista T, Harmuth T, Swertz M, Spalding D, Hoischen A, Beltran S, Graessner H, Solve RDc (2021). Solve-RD: systematic pan-European data sharing and collaborative analysis to solve rare diseases. Eur J Hum Genet.

[138] Wangler MF, Yamamoto S, Chao HT, Posey JE, Westerfield M, Postlethwait J, Hieter P, Boycott KM, Campeau PM, Bellen HJ, Members of the Undiagnosed Diseases N (2017). Model organisms facilitate rare disease diagnosis and therapeutic research. Genetics.

[139] Lehner B (2013). Genotype to phenotype: lessons from model organisms for human genetics. Nat Rev Genet.

[140] Takeshige K, Baba M, Tsuboi S, Noda T, Ohsumi Y (1992). Autophagy in yeast demonstrated with proteinase-deficient mutants and conditions for its induction. J Cell Biol.

[141] Liu W, Li L, Ye H, Chen H, Shen W, Zhong Y, Tian T, He H (2017). From Saccharomyces cerevisiae to human: the important gene co-expression modules. Biomed Rep.

[142] Sarto-Jackson I, Tomaska L (2016). How to bake a brain: yeast as a model neuron. Curr Genet.

[143] Falk J, Boubakar L, Castellani V (2019). Septin functions during neuro-development, a yeast perspective. Curr Opin Neurobiol.

[144] Biddick R, Young ET (2005). Yeast mediator and its role in transcriptional regulation. C R Biol.

[145] Lasserre JP, Dautant A, Aiyar RS, Kucharczyk R, Glatigny A, Tribouillard-Tanvier D, Rytka J, Blondel M, Skoczen N, Reynier P, Pitayu L, Rotig A, Delahodde A, Steinmetz LM, Dujardin G, Procaccio V, di Rago JP (2015). Yeast as a system for modeling mitochondrial disease mechanisms and discovering therapies. Dis Model Mech.

[146] Ruetenik A, Barrientos A (2018). Exploiting post-mitotic yeast cultures to model neurodegeneration. Front Mol Neurosci.

[147] Rapti G (2020). A perspective on C. elegans neurodevelopment: from early visionaries to a booming neuroscience research. J Neurogenet.

[148] Lai CH, Chou CY, Ch'ang LY, Liu CS, Lin W (2000). Identification of novel human genes evolutionarily conserved in Caenorhabditis elegans by comparative proteomics. Genome Res.

[149] Bessa C, Maciel P, Rodrigues AJ, Using C (2013). elegans to decipher the cellular and molecular mechanisms underlying neurodevelopmental disorders. Mol Neurobiol.

[150] Lewis EB (1978). A gene complex controlling segmentation in Drosophila. Nature.

[151] Pandey UB, Nichols CD (2011). Human disease models in Drosophila melanogaster and the role of the fly in therapeutic drug discovery. Pharmacol Rev.

[152] Frank CA (2014). Homeostatic plasticity at the Drosophila neuromuscular junction. Neuropharmacology.

[153] Coll-Tane M, Krebbers A, Castells-Nobau A, Zweier C, Schenck A. Intellectual disability and autism spectrum disorders ‘on the fly’: insights from Drosophila. Dis Model Mech. 2019;12(5):dmm039180. 10.1242/dmm.039180 PMC6550041 31088981

[154] Howe K, Clark MD, Torroja CF, Torrance J, Berthelot C, Muffato M, Collins JE, Humphray S, McLaren K, Matthews L, McLaren S, Sealy I, Caccamo M, Churcher C, Scott C, Barrett JC, Koch R, Rauch GJ, White S, Chow W, Kilian B, Quintais LT, Guerra-Assuncao JA, Zhou Y, Gu Y, Yen J, Vogel JH, Eyre T, Redmond S, Banerjee R, Chi J, Fu B, Langley E, Maguire SF, Laird GK, Lloyd D, Kenyon E, Donaldson S, Sehra H, Almeida-King J, Loveland J, Trevanion S, Jones M, Quail M, Willey D, Hunt A, Burton J, Sims S, McLay K, Plumb B, Davis J, Clee C, Oliver K, Clark R, Riddle C, Elliot D, Threadgold G, Harden G, Ware D, Begum S, Mortimore B, Kerry G, Heath P, Phillimore B, Tracey A, Corby N, Dunn M, Johnson C, Wood J, Clark S, Pelan S, Griffiths G, Smith M, Glithero R, Howden P, Barker N, Lloyd C, Stevens C, Harley J, Holt K, Panagiotidis G, Lovell J, Beasley H, Henderson C, Gordon D, Auger K, Wright D, Collins J, Raisen C, Dyer L, Leung K, Robertson L, Ambridge K, Leongamornlert D, McGuire S, Gilderthorp R, Griffiths C, Manthravadi D, Nichol S, Barker G, Whitehead S, Kay M, Brown J, Murnane C, Gray E, Humphries M, Sycamore N, Barker D, Saunders D, Wallis J, Babbage A, Hammond S, Mashreghi-Mohammadi M, Barr L, Martin S, Wray P, Ellington A, Matthews N, Ellwood M, Woodmansey R, Clark G, Cooper J, Tromans A, Grafham D, Skuce C, Pandian R, Andrews R, Harrison E, Kimberley A, Garnett J, Fosker N, Hall R, Garner P, Kelly D, Bird C, Palmer S, Gehring I, Berger A, Dooley CM, Ersan-Urun Z, Eser C, Geiger H, Geisler M, Karotki L, Kirn A, Konantz J, Konantz M, Oberlander M, Rudolph-Geiger S, Teucke M, Lanz C, Raddatz G, Osoegawa K, Zhu B, Rapp A, Widaa S, Langford C, Yang F, Schuster SC, Carter NP, Harrow J, Ning Z, Herrero J, Searle SM, Enright A, Geisler R, Plasterk RH, Lee C, Westerfield M, de Jong PJ, Zon LI, Postlethwait JH, Nusslein-Volhard C, Hubbard TJ, Roest Crollius H, Rogers J, Stemple DL (2013). The zebrafish reference genome sequence and its relationship to the human genome. Nature.

[155] Kozol RA, Abrams AJ, James DM, Buglo E, Yan Q, Dallman JE (2016). Function over form: modeling groups of inherited neurological conditions in zebrafish. Front Mol Neurosci.

[156] Saleem S, Kannan RR (2018). Zebrafish: an emerging real-time model system to study Alzheimer’s disease and neurospecific drug discovery. Cell Death Discov.

[157] de Abreu MS, Genario R, Giacomini A, Demin KA, Lakstygal AM, Amstislavskaya TG, Fontana BD, Parker MO, Kalueff AV (2020). Zebrafish as a model of neurodevelopmental disorders. Neuroscience.

[158] Breschi A, Gingeras TR, Guigo R (2017). Comparative transcriptomics in human and mouse. Nat Rev Genet.

[159] El-Khoury R, Panayotis N, Matagne V, Ghata A, Villard L, Roux JC (2014). GABA and glutamate pathways are spatially and developmentally affected in the brain of Mecp2-deficient mice. PLoS One.

[160] Shi D, Xu S, Waddell J, Scafidi S, Roys S, Gullapalli RP, McKenna MC (2012). Longitudinal in vivo developmental changes of metabolites in the hippocampus of Fmr1 knockout mice. J Neurochem.

[161] Vazquez LE, Chen HJ, Sokolova I, Knuesel I, Kennedy MB (2004). SynGAP regulates spine formation. J Neurosci Off J Soc Neurosci.

[162] Gantz SC, Ford CP, Neve KA, Williams JT (2011). Loss of Mecp2 in substantia nigra dopamine neurons compromises the nigrostriatal pathway. J Neurosci Off J Soc Neurosci.

[163] Ardhanareeswaran K, Mariani J, Coppola G, Abyzov A, Vaccarino FM (2017). Human induced pluripotent stem cells for modelling neurodevelopmental disorders. Nat Rev Neurol.

[164] Fernando MB, Ahfeldt T, Brennand KJ (2020). Modeling the complex genetic architectures of brain disease. Nat Genet.

[165] Zhao X, Bhattacharyya A (2018). Human models are needed for studying human neurodevelopmental disorders. Am J Hum Genet.

[166] Frega M, van Gestel SH, Linda K, van der Raadt J, Keller J, Van Rhijn JR, et al. Rapid neuronal differentiation of induced pluripotent stem cells for measuring network activity on Micro-electrode arrays. J Vis Exp. 2017;(119):54900. 10.3791/54900. 10.3791/54900 PMC5407693 28117798

[167] Pasca AM, Sloan SA, Clarke LE, Tian Y, Makinson CD, Huber N, Kim CH, Park JY, O'Rourke NA, Nguyen KD, Smith SJ, Huguenard JR, Geschwind DH, Barres BA, Pasca SP (2015). Functional cortical neurons and astrocytes from human pluripotent stem cells in 3D culture. Nat Methods.

[168] Sabitha KR, Shetty AK, Upadhya D (2021). Patient-derived iPSC modeling of rare neurodevelopmental disorders: molecular pathophysiology and prospective therapies. Neurosci Biobehav Rev.

[169] Ortuno-Costela MDC, Cerrada V, Garcia-Lopez M, Gallardo ME (2019). The challenge of bringing iPSCs to the patient. Int J Mol Sci.

[170] Liang G, Zhang Y (2013). Genetic and epigenetic variations in iPSCs: potential causes and implications for application. Cell Stem Cell.

[171] Fell CW, Nagy V (2021). Cellular models and high-throughput screening for Genetic causality of intellectual disability. Trends Mol Med.

[172] Vierbuchen T, Ostermeier A, Pang ZP, Kokubu Y, Sudhof TC, Wernig M (2010). Direct conversion of fibroblasts to functional neurons by defined factors. Nature.

[173] Tanabe K, Ang CE, Chanda S, Olmos VH, Haag D, Levinson DF, Sudhof TC, Wernig M (2018). Transdifferentiation of human adult peripheral blood T cells into neurons. Proc Natl Acad Sci U S A.

[174] Ran FA, Hsu PD, Wright J, Agarwala V, Scott DA, Zhang F (2013). Genome engineering using the CRISPR-Cas9 system. Nat Protoc.

[175] Adli M (2018). The CRISPR tool kit for genome editing and beyond. Nat Commun.

[176] Ben Jehuda R, Shemer Y, Binah O (2018). Genome editing in induced pluripotent stem cells using CRISPR/Cas9. Stem Cell Rev Rep.

[177] Alkan F, Wenzel A, Anthon C, Havgaard JH, Gorodkin J (2018). CRISPR-Cas9 off-targeting assessment with nucleic acid duplex energy parameters. Genome Biol.

[178] Anzalone AV, Randolph PB, Davis JR, Sousa AA, Koblan LW, Levy JM, Chen PJ, Wilson C, Newby GA, Raguram A, Liu DR (2019). Search-and-replace genome editing without double-strand breaks or donor DNA. Nature.

[179] Kampmann M (2018). CRISPRi and CRISPRa screens in mammalian cells for precision biology and medicine. ACS Chem Biol.

[180] Fellner A, Ruhrman-Shahar N, Orenstein N, Lidzbarsky G, Shuldiner AR, Gonzaga-Jauregui C, Brown-Shalev H, Hagari-Bechar O, Bazak L, Basel-Salmon L (2021). The role of phenotype-based search approaches using public online databases in diagnostics of Mendelian disorders. Genet Med.

[181] Kohler S, Schulz MH, Krawitz P, Bauer S, Dolken S, Ott CE, Mundlos C, Horn D, Mundlos S, Robinson PN (2009). Clinical diagnostics in human genetics with semantic similarity searches in ontologies. Am J Hum Genet.

[182] Biesecker LG, Adam MP, Alkuraya FS, Amemiya AR, Bamshad MJ, Beck AE, Bennett JT, Bird LM, Carey JC, Chung B, Clark RD, Cox TC, Curry C, Dinulos MBP, Dobyns WB, Giampietro PF, Girisha KM, Glass IA, Graham JM, Gripp KW, Haldeman-Englert CR, Hall BD, Innes AM, Kalish JM, Keppler-Noreuil KM, Kosaki K, Kozel BA, Mirzaa GM, Mulvihill JJ, Nowaczyk MJM, Pagon RA, Retterer K, Rope AF, Sanchez-Lara PA, Seaver LH, Shieh JT, Slavotinek AM, Sobering AK, Stevens CA, Stevenson DA, Tan TY, Tan WH, Tsai AC, Weaver DD, Williams MS, Zackai E, Zarate YA (2021). A dyadic approach to the delineation of diagnostic entities in clinical genomics. Am J Hum Genet.

[183] Schaefer GB, Mendelsohn NJ, Professional P, Guidelines C (2013). Clinical genetics evaluation in identifying the etiology of autism spectrum disorders: 2013 guideline revisions. Genet Med.

[184] Manning M, Hudgins L, Professional P, Guidelines C (2010). Array-based technology and recommendations for utilization in medical genetics practice for detection of chromosomal abnormalities. Genet Med.

[185] Manickam K, McClain MR, Demmer LA, Biswas S, Kearney HM, Malinowski J, Massingham LJ, Miller D, Yu TW, Hisama FM, Directors ABo (2021). Exome and genome sequencing for pediatric patients with congenital anomalies or intellectual disability: an evidence-based clinical guideline of the American College of Medical Genetics and Genomics (ACMG). Genet Med.

[186] de Brouwer AP, Yntema HG, Kleefstra T, Lugtenberg D, Oudakker AR, de Vries BB, van Bokhoven H, Van Esch H, Frints SG, Froyen G, Fryns JP, Raynaud M, Moizard MP, Ronce N, Bensalem A, Moraine C, Poirier K, Castelnau L, Saillour Y, Bienvenu T, Beldjord C, des Portes V, Chelly J, Turner G, Fullston T, Gecz J, Kuss AW, Tzschach A, Jensen LR, Lenzner S, Kalscheuer VM, Ropers HH, Hamel BC (2007). Mutation frequencies of X-linked mental retardation genes in families from the EuroMRX consortium. Hum Mutat.

[187] van Karnebeek CD, Jansweijer MC, Leenders AG, Offringa M, Hennekam RC (2005). Diagnostic investigations in individuals with mental retardation: a systematic literature review of their usefulness. Eur J Hum Genet.

[188] Tarpey PS, Smith R, Pleasance E, Whibley A, Edkins S, Hardy C, O’Meara S, Latimer C, Dicks E, Menzies A, Stephens P, Blow M, Greenman C, Xue Y, Tyler-Smith C, Thompson D, Gray K, Andrews J, Barthorpe S, Buck G, Cole J, Dunmore R, Jones D, Maddison M, Mironenko T, Turner R, Turrell K, Varian J, West S, Widaa S, Wray P, Teague J, Butler A, Jenkinson A, Jia M, Richardson D, Shepherd R, Wooster R, Tejada MI, Martinez F, Carvill G, Goliath R, de Brouwer AP, van Bokhoven H, Van Esch H, Chelly J, Raynaud M, Ropers HH, Abidi FE, Srivastava AK, Cox J, Luo Y, Mallya U, Moon J, Parnau J, Mohammed S, Tolmie JL, Shoubridge C, Corbett M, Gardner A, Haan E, Rujirabanjerd S, Shaw M, Vandeleur L, Fullston T, Easton DF, Boyle J, Partington M, Hackett A, Field M, Skinner C, Stevenson RE, Bobrow M, Turner G, Schwartz CE, Gecz J, Raymond FL, Futreal PA, Stratton MR (2009). A systematic, large-scale resequencing screen of X-chromosome coding exons in mental retardation. Nat Genet.

[189] Hamdan FF, Gauthier J, Araki Y, Lin DT, Yoshizawa Y, Higashi K, Park AR, Spiegelman D, Dobrzeniecka S, Piton A, Tomitori H, Daoud H, Massicotte C, Henrion E, Diallo O, Shekarabi M, Marineau C, Shevell M, Maranda B, Mitchell G, Nadeau A, D’Anjou G, Vanasse M, Srour M, Lafreniere RG, Drapeau P, Lacaille JC, Kim E, Lee JR, Igarashi K, Huganir RL, Rouleau GA, Michaud JL, Group SD (2011). Excess of de novo deleterious mutations in genes associated with glutamatergic systems in nonsyndromic intellectual disability. Am J Hum Genet.

[190] Najmabadi H, Motazacker MM, Garshasbi M, Kahrizi K, Tzschach A, Chen W, Behjati F, Hadavi V, Nieh SE, Abedini SS, Vazifehmand R, Firouzabadi SG, Jamali P, Falah M, Seifati SM, Gruters A, Lenzner S, Jensen LR, Ruschendorf F, Kuss AW, Ropers HH (2007). Homozygosity mapping in consanguineous families reveals extreme heterogeneity of non-syndromic autosomal recessive mental retardation and identifies 8 novel gene loci. Hum Genet.

[191] Kuss AW, Garshasbi M, Kahrizi K, Tzschach A, Behjati F, Darvish H, Abbasi-Moheb L, Puettmann L, Zecha A, Weissmann R, Hu H, Mohseni M, Abedini SS, Rajab A, Hertzberg C, Wieczorek D, Ullmann R, Ghasemi-Firouzabadi S, Banihashemi S, Arzhangi S, Hadavi V, Bahrami-Monajemi G, Kasiri M, Falah M, Nikuei P, Dehghan A, Sobhani M, Jamali P, Ropers HH, Najmabadi H (2011). Autosomal recessive mental retardation: homozygosity mapping identifies 27 single linkage intervals, at least 14 novel loci and several mutation hotspots. Hum Genet.

[192] de Vries BB, Pfundt R, Leisink M, Koolen DA, Vissers LE, Janssen IM, Reijmersdal S, Nillesen WM, Huys EH, Leeuw N, Smeets D, Sistermans EA, Feuth T, van Ravenswaaij-Arts CM, van Kessel AG, Schoenmakers EF, Brunner HG, Veltman JA (2005). Diagnostic genome profiling in mental retardation. Am J Hum Genet.

[193] James KN, Clark MM, Camp B, Kint C, Schols P, Batalov S, Briggs B, Veeraraghavan N, Chowdhury S, Kingsmore SF (2020). Partially automated whole-genome sequencing reanalysis of previously undiagnosed pediatric patients can efficiently yield new diagnoses. NPJ Genomic Med.

[194] Costain G, Jobling R, Walker S, Reuter MS, Snell M, Bowdin S, Cohn RD, Dupuis L, Hewson S, Mercimek-Andrews S, Shuman C, Sondheimer N, Weksberg R, Yoon G, Meyn MS, Stavropoulos DJ, Scherer SW, Mendoza-Londono R, Marshall CR (2018). Periodic reanalysis of whole-genome sequencing data enhances the diagnostic advantage over standard clinical genetic testing. Eur J Hum Genet.

[195] Won D, Kim SH, Kim B, Lee ST, Kang HC, Choi JR (2020). Reanalysis of genomic sequencing results in a clinical laboratory: advantages and limitations. Front Neurol.

[196] Rhoads A, Au KF (2015). PacBio sequencing and its applications. Genomics Proteomics Bioinformatics.

[197] Muller CA, Boemo MA, Spingardi P, Kessler BM, Kriaucionis S, Simpson JT, Nieduszynski CA (2019). Capturing the dynamics of genome replication on individual ultra-long nanopore sequence reads. Nat Methods.

[198] Mantere T, Kersten S, Hoischen A (2019). Long-read sequencing emerging in medical genetics. Front Genet.

[199] Houge G, Laner A, Cirak S, de Leeuw N, Scheffer H, den Dunnen JT. Stepwise ABC system for classification of any type of genetic variant. Eur J Hum Genet. 2021; Epub ahead of print. 10.1038/s41431-021-00903-z PMC8821602 33981013

